# Inversions maintain differences between migratory phenotypes of a songbird

**DOI:** 10.1038/s41467-023-36167-y

**Published:** 2023-01-27

**Authors:** Max Lundberg, Alexander Mackintosh, Anna Petri, Staffan Bensch

**Affiliations:** 1 Department of Biology, Lund University, Lund, Sweden; 2 Institute of Ecology and Evolution, University of Edinburgh, Edinburgh, UK; 3 Science for Life Laboratory, Uppsala Genome Center, Uppsala University, Uppsala, Sweden

**Keywords:** Evolutionary genetics, Structural variation, Molecular ecology, Animal migration

## Abstract

Structural rearrangements have been shown to be important in local adaptation and speciation, but have been difficult to reliably identify and characterize in non-model species. Here we combine long reads, linked reads and optical mapping to characterize three divergent chromosome regions in the willow warbler *Phylloscopus trochilus*, of which two are associated with differences in migration and one with an environmental gradient. We show that there are inversions (0.4–13 Mb) in each of the regions and that the divergence times between inverted and non-inverted haplotypes are similar across the regions (~1.2 Myrs), which is compatible with a scenario where inversions arose in either of two allopatric populations that subsequently hybridized. The improved genomes allow us to detect additional functional differences in the divergent regions, providing candidate genes for migration and adaptations to environmental gradients.

## Abstract (web summary)

Rearrangements in the genome are important for local adaptation and speciation but are often difficult to identify reliably. Here the authors show that rearrangements underlie large chromosome regions that separate differentially migratory willow warblers.

## Introduction

Loci underlying local adaptation and speciation have been found to be concentrated in inversions across many species of animals and plants^1^. By capturing co-adapted variants at linked loci, inversions facilitate the formation of supergenes, where complex phenotypes are inherited as if they were encoded by a single gene^2^. For example, inversion polymorphisms have been associated with different mating types in birds^3,4^, social polymorphisms in insects^5^ and differences in migratory phenotypes in fish^6,7^. However, for non-model species, larger inversions have been difficult to reliably identify and characterize, as breakpoints often coincide with repeat-rich genomic regions that are difficult to assemble, particularly with short-read sequencing technologies^8^. Overcoming these challenges will be important for broadening our understanding of local adaptation and speciation.

The willow warbler *Phylloscopus trochilus* is represented by two differentially migrating populations in Europe^9,10^. The southern migratory phenotype (ssp. *trochilus*) occurs in Western Europe and migrates to Western Africa. The northern migratory phenotype (ssp. *acredula*) breeds in Northern Scandinavia and Eastern Europe and winters in Eastern or Southern Africa. The subspecies are otherwise morphologically and ecologically similar^11,12^.

In the most comprehensive genetic study of the willow warbler to date, Lundberg et al.^13^ assembled a draft genome based on short-read data and used whole-genome resequencing and a customized 4000 SNP array to explore genetic differences between the migratory phenotypes. The vast majority of variants that were highly differentiated between the migratory phenotypes were located in three divergent regions on chromosomes 1, 3, and 5. Variation in the regions on chromosomes 1 and 5 was strongly associated with migratory phenotypes, while the region on chromosome 3 showed a stronger association with latitude and altitude. The clearly delimited plateaus of high genetic differentiation and the apparent lack of recombination between divergent southern and northern haplotypes in these regions suggested the presence of inversion polymorphisms. However, no inversion breakpoints could be identified, presumably because they were located in repeat rich regions. In addition, the two migration-linked regions on chromosomes 1 and 5, were split into two and ten scaffolds, respectively, making it difficult to know if the gene order within these regions is different from what is found in other birds.

**Fig. 1:**
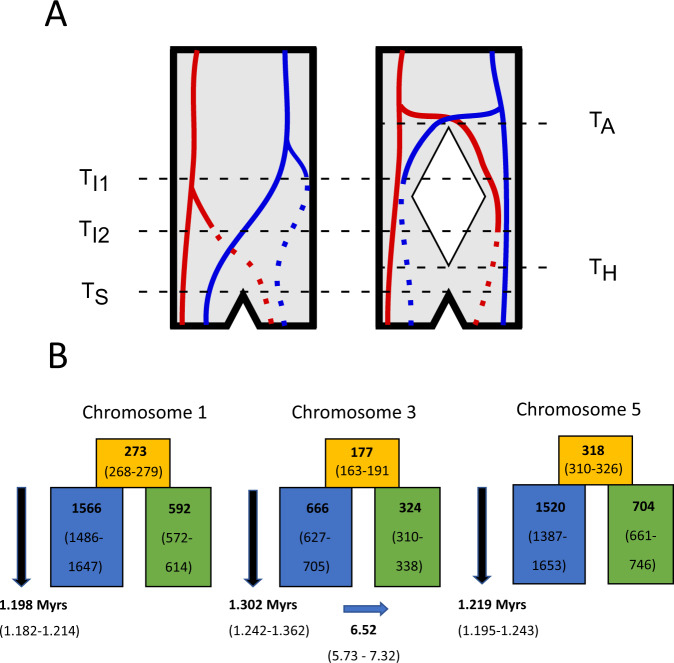
Evolutionary history of inversions. **A** Alternative population scenarios for two inversions (lines changing from solid to dotted) appearing at separate loci (red and blue) at timepoints T_I1_ and T_I2_, respectively. In the left scenario, the inversions appear as polymorphisms within a single ancestral population and eventually get sorted into one of two subspecies (T_S_). The divergence times of the inverted and non-inverted haplotypes will, in this case, reflect the timepoints of the inversion events. In the right scenario, an ancestral population splits into two allopatric populations (T_A_) and inversions appear in one of them. The two populations later come into secondary contact and merge (T_H_), before the inversions get sorted into one of two subspecies (T_S_). In this scenario, the divergence times of the inverted and non-inverted haplotypes for the two loci will be similar as they do not reflect the time of the inversion events, but the time of the ancestral population split (T_A_). **B** Population modeling parameter estimates (maximum composite likelihood with 95CI) for the three divergent regions in the willow warbler. Orange, blue, and green boxes refer to ancestral, northern and southern effective population sizes, respectively, with numbers given in units of 10^3^. For chromosome 3, simulations confirmed a better fit for a model including migration (IM_2_), and in this case the blue arrow indicates the direction of migration with the rate given in units of 10^−7^. All three divergent regions have similar split times (~1.2 Myrs), as expected from the right scenario in (**A**).

In this study, we use long-read sequencing, linked-read sequencing, optical mapping and RNAseq to create more complete, contiguous and well-annotated genome assemblies of a southern and a northern willow warbler. The new genome assemblies allow us to explore the structural organization of the divergent chromosome regions in each subspecies, to examine if additional highly differentiated regions between the migratory phenotypes might reside in parts of the genome not included in the previous short-read genome assembly, and to assess functional consequences of highly differentiated variants. We also use long read sequencing to assemble a genome for the chiffchaff *Phylloscopus collybita* and compare this to the willow warbler assemblies to gain information about the evolutionary histories of the divergent regions. Finally, we fit models of population divergence using the information within the blockwise site frequency spectrum (bSFS)^14^. This approach, adapted from Lohse et al.^15^, estimates the ancestral effective population size as well as the rate of migration, and so provides more accurate estimates of population divergence time than summary statistics where these parameters are assumed or ignored. If the divergent haplotypes in each of the regions, which are presently associated with each subspecies, are associated with inversions that arose independently within a single ancestral population, we would have no expectation of synchronized divergence times between regions (Fig. 1). An alternative scenario is that the divergent haplotypes are a consequence of an ancient hybridization event, which has previously been hypothesized to explain the diversity in the extant willow warbler populations^16^. In this scenario, northern and southern haplotypes were unique to either of the hybridizing populations and were protected from recombining with each other through inversions, whereas the rest of the genome was homogenized through gene flow. In this case, we expect the divergence times to be similar across the regions (Fig. 1) because they would not represent the actual inversion events, but rather the time of the ancient population split^17^.

## Results

### Genome assemblies

Using a combination of long-read sequencing, linked-read sequencing and optical mapping, we obtained highly contiguous de novo assemblies for both a southern and a northern willow warbler, which contained 547 scaffolds with an N50 of 34 Mb and 496 scaffolds with an N50 of 17 Mb, respectively (Supplementary Table 1). These contiguity metrics represent an order of magnitude improvement compared to those of a previous short-read willow warbler genome assembly^13^ and are similar to or exceed those of other bird assemblies combining long reads and optical mapping data^18,19^. Using HiFi long-read sequencing, we generated a de novo assembly of a chiffchaff consisting of 517 contigs with an N50 of 28 Mb (Supplementary Table 1). The quality of the assemblies was also verified by the presence of a high percentage of complete and a low percentage of duplicated single-copy bird orthologues (94.1–95.2% and 1.1–1.5% of 4915 targeted genes, respectively, Supplementary Table 1).

The southern willow warbler, the northern willow warbler and the chiffchaff assembly contained 21.0, 14.8, and 21.2% annotated repeats, respectively, compared to 9.7% in the previous willow warbler assembly (Supplementary Table 2). The southern assembly was annotated with 22,757 protein-coding genes based on a combination of willow warbler RNAseq data (Supplementary Table 3) and protein data from other bird species.

### Differences between subspecies

We explored genetic differentiation between the subspecies by mapping whole-genome resequencing data of 11 southern and 11 northern willow warblers (Supplementary Table 4) to the southern assembly. The weighted average F_ST_ between the northern and southern samples for 45 million bi-allelic SNPs was 0.006 and the mean weighed average F_ST_ across 10 kb windows was also 0.006. Only including variants with a minor allele frequency (MAF) of at least 0.1 (12 million), the corresponding values were 0.01 and 0.007, respectively. The number of highly-differentiated variants was extremely small, with almost all located in the previously identified divergent chromosome regions (Supplementary Table 5). For example, of 11,855 variants with F_ST_ ≥ 0.7, only 140 were found outside of the three regions. The majority (84%) of these 140 variants were found on nine scaffolds (median size: 3,699,632 bp, range: 511,299–7,950,085 bp) that could not be confidently assigned to specific chromosomes in the chicken *Gallus gallus*, zebra finch *Taeniopygia guttata* or collared flycatcher *Ficedula albicollis* genomes. These scaffolds contained a high proportion of repetitive sequences (87–95 % of the ungapped length), a high GC content (50–51%) and mostly olfactory receptor genes, although it is unclear to what extent these genes are functional. Additionally, coverage on these scaffolds was generally lower in the northern than in the southern resequenced samples (Supplementary Fig. 1) and similar scaffolds were also found in the northern assembly, but could only be partially aligned to scaffolds in the southern genome. This suggests that these scaffolds represent genomic regions that show different higher-order repeat organization between the subspecies.

### Chromosome 1 region

**Fig. 2:**
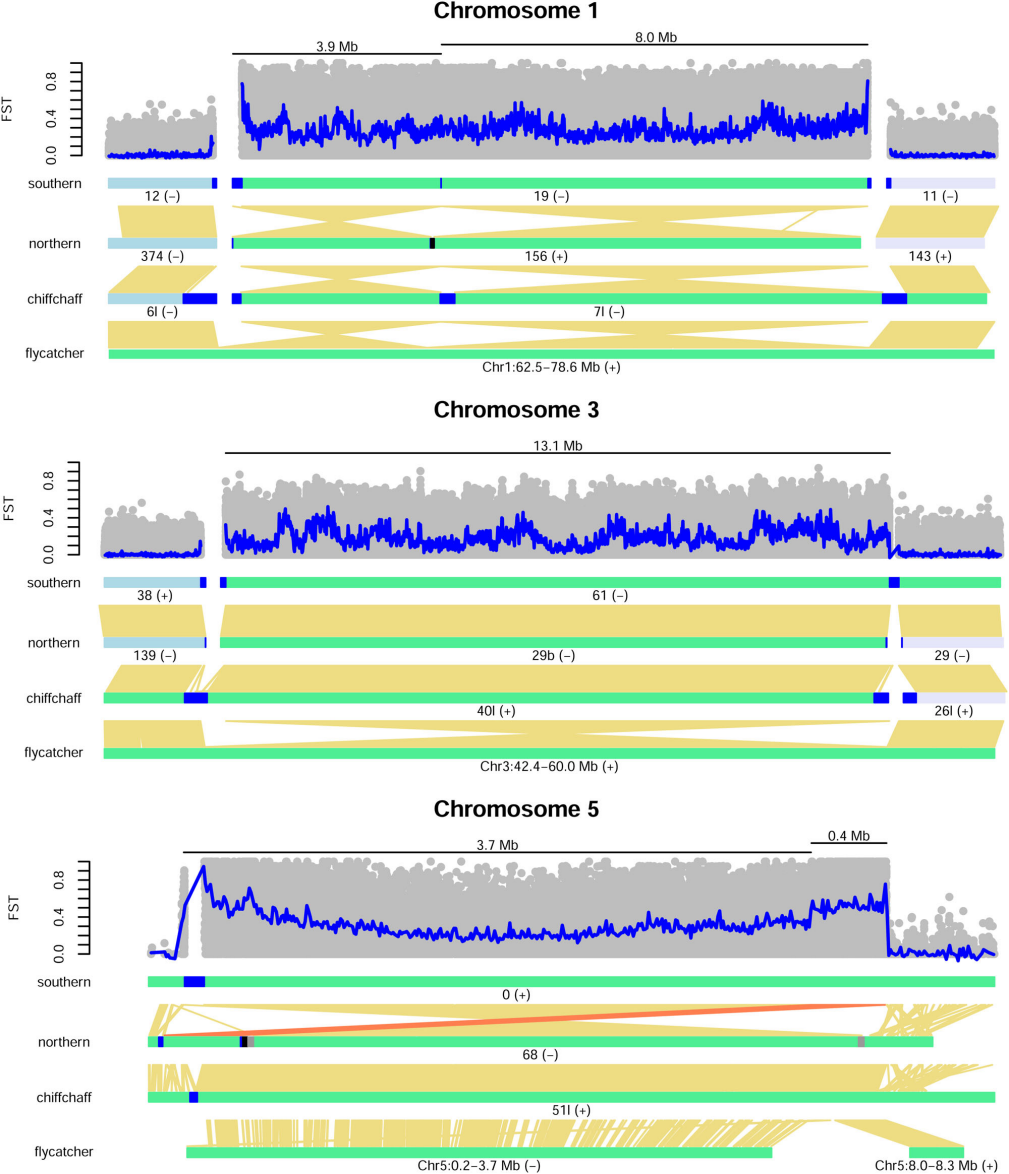
Divergent regions on chromosome 1, 3, and 5 in the southern assembly. The top panel shows genetic differentiation (F_ST_) between 11 resequenced samples from each subspecies for variants with a minor allele frequency (MAF) ≥ 0.1, with blue lines representing a weighted average for MAF ≥ 0.1 bi-allelic SNPs in 10 kb non-overlapping windows. Below, scaffolds or chromosomes (chr) in each assembly matching the divergent regions are shown as light green rectangles (with ID and plotted orientation) and predicted upstream and downstream scaffolds as light blue and light purple rectangles, respectively. Blue segments on scaffolds show the location of large tandem arrays at the ends or breakpoint regions, black segments represent gaps and gray segments the location of 31 kb duplicated intervals in the chromosome 5 region on the northern assembly. Yellow lines above scaffolds represent 1:1 alignment intervals (≥2 kb) to scaffolds in the southern assembly. For chromosome 5, the second inversion interval has been highlighted in orange to improve visibility. The zebra finch genome shows the same major structural differences as seen in the flycatcher genome and has, therefore, not been included.

In the southern assembly, the highly differentiated region on chromosome 1 was assembled into an 11.9 Mb (gap free) scaffold (Scaffold19, Fig. 2). The start and end of the scaffold contain 49 and 174 kb arrays, respectively, of a 413 bp tandem repeat. The divergent region could not be joined with other parts of chromosome 1, but the ends of the predicted adjacent scaffolds, based on the flycatcher and zebra finch genomes, both contain arrays (67 and 70 kb) of the same tandem repeat (Fig. 2), which likely explains why this region is difficult to completely assemble even with HiFi long reads and optical mapping data. In the northern assembly, the divergent region was assembled into a similarly sized scaffold (11.7 Mb), which contained a 58 kb gap surrounded by tandem repeats. The southern scaffold lacked a long tandem repeat array in the interval corresponding to the gap region, but a 270 kb array was present in the chiffchaff assembly. In the chiffchaff, the divergent region was connected at one end with the part of chromosome 1 predicted from the flycatcher and zebra finch. At this connection, there was a 440 kb tandem repeat array.

The southern de novo assembly differed from the other genomes by the presence of two tandemly arranged inversions in the divergent region, which are 3.7 Mb and 7.9 Mb (Fig. 2). The shared breakpoint interval coincides with the more centrally located tandem repeat array in the northern willow warbler and the chiffchaff, but in the southern assembly, there is only a 1.5 kb interval of the same repeat. The difference in breakpoints between the southern and the northern sample was also supported by barcode coverage of linked reads (Supplementary Fig. 2) and by optical maps, where a translocation from the start to the end of the divergent scaffold in the northern assembly was detected in the southern sample.

Genetic differentiation between the 11 resequenced samples from each subspecies was high across the entire region (mean weighted F_ST_ in 10 kb windows for bi-allelic SNPs with MAF ≥ 0.1: 0.28), but showed prominent peaks at the start and at the end (Fig. 2).

### Chromosome 3 region

In the southern assembly, the highly differentiated region on chromosome 3 (13.1 Mb) was located at the end of a 69.3 Mb scaffold (Scaffold61) and shows a reverse orientation compared to the flycatcher and zebra finch (Fig. 2). In between the divergent region and the remainder of the scaffold was a 184 kb tandem repeat array of the same type as on chromosome 1. The same type of tandem repeat array was also found at the opposite end of the divergent region (two intervals of 12 and 78 kb) and at the end of the predicted adjacent scaffold (94 kb, Scaffold38, Fig. 2). As in the region on chromosome 1, we did not find any evidence of tandem repeat arrays in the zebra finch or flycatcher. In the northern assembly, the divergent region was contained within a 13.2 Mb scaffold (Scaffold29b) that could not be reliably scaffolded with other undifferentiated parts of chromosome 3. The start of the scaffold contained an 8 kb tandem repeat array and the end contained a 673 bp interval of the same tandem repeat, which was followed by a 296 bp LTR/ERVL repeat. In the chiffchaff assembly, the divergent region was also found in the same (reverse) orientation as in the southern willow warbler, but was joined with other parts of the chromosome at the other end (contig ptg000040l, Fig. 2). This join was associated with a 444 kb tandem repeat array and, similar to the willow warblers, tandem repeat arrays were also present at the other end of the divergent region interval (279 kb) and at the end of the predicted adjacent contig (252 kb, ptg000026l).

At the end of the southern scaffold (Scaffold61, start of the region in Fig. 2), there was a breakpoint difference between the willow warbler subspecies suggested by long read alignments (Supplementary Fig. 3). Over this interval, reads from the northern willow warbler cannot be properly aligned beyond the start of the repeat array and this pattern was also observed for the chiffchaff reads. The corresponding region in the chiffchaff assembly shows a different structural configuration compared to the southern willow warbler, where an additional interval of 9.7 kb consisting mainly of LTR/ERVL repeats exists between a 765 and a 444 kb tandem repeat array. In contrast to the northern willow warbler, long reads from the southern willow warbler do not align within this region (Supplementary Fig. 3). This suggests that the northern willow warbler and chiffchaff share a more ancestral configuration within the region, and that the inversion observed in the chiffchaff may be the result of an independent event compared to the southern willow warbler.

Highly differentiated variants were present across the entire differentiated region (mean weighted F_ST_ in 10 kb windows for bi-allelic SNPs with MAF ≥ 0.1: 0.19), but as opposed to the region on chromosome 1, we did not see any clear breakpoint effect (Fig. 2).

### Chromosome 5 region

In the southern assembly, the highly differentiated region on chromosome 5 (4.1 Mb) is part of a 67 Mb scaffold (Scaffold0) that covers most of the chromosome (Fig. 2). Within the divergent interval, the southern willow warbler is mostly collinear with the flycatcher and the zebra finch. On each side of the divergent interval, there are repeat-rich regions that could not be confidently aligned to the other species. In the northern assembly, the divergent interval is embedded within a 4.6 Mb scaffold (Scaffold68, Fig. 2). Compared to the southern willow warbler, the northern willow warbler has a 3.7 Mb inverted and a 0.4 Mb collinear but translocated interval, which are separated by a gap of 41 kb (Fig. 2). This gap is surrounded by repeats that are found in tandem at the end of the scaffold and form a single 116 kb array at the start of the region in the southern assembly. Furthermore, the inverted interval in the northern assembly is surrounded by 31 kb segmental duplications that show 94% identity to each other (Fig. 2). The duplicated interval is rich in repeats and contains a truncated copy of the Nucleolar pre-ribosomal-associated protein 1 (*URB1*) gene, which in the southern assembly is found as a near complete copy upstream of the region.

Based on the mix of inverted and collinear intervals between the willow warbler subspecies, a possible scenario is that the entire differentiated interval was first inverted in the northern subspecies and a second smaller inversion event restored the order at the end. In the chiffchaff assembly, the divergent region is embedded in a 6.8 Mb contig (ptg000051l) and shows the same orientation as in the southern willow warbler (Fig. 2). This suggests that the northern willow warbler has the derived orientation. Structural differences between the subspecies in this region were further supported by alignments of linked reads, where the longranger wgs pipeline detected the breakpoint difference at ~ 4.0 Mb in the northern scaffold (Scaffold68) and a deletion in the northern sample coinciding with the tandem repeat region at the start of the region in the southern scaffold (Scaffold0). Furthermore, linked read barcode coverage was lower for the northern sample than the other two samples in the breakpoint regions on the southern scaffold (Supplementary Fig. 2), although the pattern was not as clear around the tandem repeat region at the start due to the overall low coverage. Finally, structural differences between the subspecies were evident when hybridizing the optical map of the northern sample to the assembly of the southern sample (Supplementary Fig. 4).

Genetic differentiation between resequenced southern and northern birds was high (mean weighted F_ST_ in 10 kb windows for bi-allelic SNPs with MAF ≥ 0.1: 0.32) within the entire region, but was on average higher in the 0.4 Mb translocated interval (Fig. 2). As in the case of the region on chromosome 1, there was a breakpoint effect in genetic differentiation, with particularly high values towards the start and the end of the region.

### The age and demography of divergent regions

We used two high-coverage (24–44×) resequenced samples of each subspecies to estimate the population divergence time between the subspecies in each of the divergent regions (Supplementary Table 4). First, we calculated net divergence (*d*_a_)^20^ and found similar values across the three regions (Supplementary Table 6). Using a germline mutation rate estimated from the collared flycatcher^21^, and assuming a generation time of 1.7 years^11^, these values correspond to divergence times of 512, 544, and 539 kyr for the regions on chromosomes 1, 3, and 5, respectively. As a complementary, but not independent measurement of divergence, we calculated relative node depth (RND)^22^ using a dusky warbler *Phylloscopus fuscatus* (Supplementary Table 4) as an outgroup. The RND estimates were similar across the regions (0.35–0.41, Supplementary Table 6) and suggest that divergence arose far more recently than the shared ancestor of the willow warbler and dusky warbler.

Divergence-based methods are expected to lead to underestimated divergence times if there has been gene flow occurring between populations. Furthermore, if diversity in the ancestral population was much larger or smaller than in the contemporary populations, d_a_ would be biased upwards or downwards, respectively. To overcome these caveats, and obtain more robust estimates of population divergence, we fitted demographic models involving divergence and gene flow between two populations for each divergent region. We used the software gIMble^23^, which leverages information within the blockwise site frequency spectrum (bSFS)^14^ to compare the support (composite likelihoods (CLs)) for different demographic models and parameters.

For the divergent regions on chromosomes 1 and 5 the best fitting model (i.e., the highest CL) was the IM_1_ model. In this model, the population split is followed by a constant rate of migration (m_e_) from the southern population to the northern population forwards in time. For the divergent region on chromosome 3 the greatest CL was found using the IM_2_ model, where migration occurs in the opposite direction to IM_1_. However, the IM models are expected to always achieve a greater CL than strict isolation (SI) models because they include an additional parameter, the rate of migration. Additionally, the maximum composite likelihood (MCL) estimates of migration rate were small (4.45 × 10^−7^, 6.53 × 10^−7^ and 3.46 × 10^−7^ for chromosomes 1, 3, and 5, respectively), which, although consistent with limited recombination within inversions, suggests that the IM model may not fit significantly better than SI. Therefore, to test whether the IM models gave a significant improvement in CL, we simulated replicates under the optimized SI parameters for each divergent region and recorded the difference in CL between the IM and SI model. For the regions on chromosomes 1 and 5 we found that the improvement in CL between models was entirely consistent with a history of strict isolation (Supplementary Fig. 5). By contrast, the improvement in CL observed for the chromosome 3 region is greater than we would expect if there had been no migration. Hereafter we present and discuss parameter estimates from the simpler SI model for the chromosome 1 and 5 regions and from the IM_2_ model for the chromosome 3 region (Fig. 1).

The MCL parameter estimates suggest that the divergence time within each divergent region is around 1.2 Myr and thus far greater than was estimated by our d_a_ calculation. Effective population sizes were consistently estimated to be higher in the northern willow warblers and those of the ancestral populations smaller than in the contemporary populations. The similar parameter estimates among the three regions, especially divergence time (1.20–1.30 Myrs), suggests that they have a shared demographic history and supports a scenario where inversions happened in allopatric populations.

Consistent with the idea that almost all of the genetic differentiation between these subspecies is confined to only three regions, the MCL estimates of population divergence time presented above are an order of magnitude greater than analogous estimates over the rest of the genome (Supplementary Table 7). However, the distribution of coalescence times outside of the divergent regions may still contain information about the demographic history of these subspecies. For example, a sustained period of population structure will result in a reduced coalescence rate (increased N_e_) until populations become admixed again. To test whether the coalescence rate over time follows such a pattern, we used the sequentially Markovian coalescent (SMC) implemented in MSMC2^24^. For each sample, N_e_ was estimated to have been highest during the Pleistocene, peaking at ~450 kya, and lower in more recent (<200 kya) and distant (>2 Mya) past (Supplementary Fig. 6). Although there are multiple explanations for this pattern, it is at least consistent with the possibility that a period of allopatry, beginning 1.2 Mya and ending <450 kya, is the reason that different divergent regions have a shared demographic history.

We also calculated several population summary statistics to infer demographic effects, such as bottlenecks. In the divergent regions, particularly on chromosomes 1 and 5, southern willow warblers had an overall lower nucleotide diversity, higher Tajima’s D and a higher abundance of high-frequency derived alleles than northern willow warblers (Supplementary Figs. 7, 8). Similarly, a haplotype-based analysis (XP-nsl) in the divergent regions generally suggested extended regions of low diversity in the southern samples compared to the northern samples (Supplementary Fig. 9).

### Functional differences in the divergent regions

With the improved assembly and annotation, we examined potential functional differences between the subspecies in the three chromosome regions. None of the breakpoint intervals overlapped with or were very close to an annotated functional protein-coding gene (range: 1.5–71.2 kb, Supplementary Table 8). We also explored whether any SNPs or short indels with high differentiation (F_ST_ ≥ 0.7) between southern and northern homozygotes were predicted to have a moderate to high impact on protein-coding genes. Across the three regions, we found 73 nonsynonymous mutations and an in-frame insertion located in 46 genes (Supplementary Table 9). We additionally found one frameshift mutation in general transcription factor IIIA (*GTF3A*), which is located within the divergent region on chromosome 1. This change, which represents a derived deletion in the northern subspecies, modifies four amino acids at the end and further extends the protein with three amino acids. Although the genes with protein-coding changes were functionally diverse, some shared more specific functions. In particular, nonsynonymous mutations were found in three fatty acid desaturase genes (*FADS2*, *FADS1L1*, *FADS1L2*) that are located in tandem in the second differentiated interval on chromosome 5. Two of these genes (*FADS2*, *FADS1L2*) are also annotated as involved in “oxidation-reduction process” together with cytochrome b5 reductase 2 (*CYB5R2*) and gamma-butyrobetaine hydroxylase 1 (*BBOX1*), also on chromosome 5, and crystallin lambda 1 (*CRYL1*) on chromosome 1.

We also searched for highly differentiated structural variants between southern and northern samples in the divergent regions. We detected 31 deletions (51–2934 bp), 24 insertions (55–1511 bp) and two duplications (52–110 bp) that had a F_ST_ ≥ 0.7 between southern and northern homozygotes in the divergent regions. The majority of the structural variants (35/57) were located outside of the annotated genes with a median distance of 47 kb and only one variant overlapped exons of protein-coding genes: a 207 bp insertion in the 3’UTR of the Stomatin Like 3 (*STOML3*) gene located in the region on chromosome 1.

Finally, we explored if there were any signs of recent positive selection in genes within the divergent regions. A strong signal was found in one of the introns of the Spondin-1 (*SPON1*) gene, which is located at the start of the chromosome 5 region (Supplementary Figs. 9, 10). Here, we found a high proportion of SNPs that were northern outliers for XP-nsl, as well as high Sweepfinder2 CLR values and reduced nucleotide diversity in northern samples.

## Discussion

Using highly contiguous genomes, we have demonstrated that the divergent regions separating the two willow warbler subspecies are associated with structural rearrangements. We also corroborated the results of Lundberg et al.^13^ by finding that virtually all the highly differentiated SNPs and indels between the subspecies are located in these regions. However, the long-read sequencing and optical mapping data enabled us to identify additional differences in previously overlooked repeat-rich scaffolds that may represent more large-scale structural differences between the subspecies. Due to their high repeat content, we failed to confidently assign these scaffolds to specific chromosomes in other bird species, nor determine whether the similar regions in the northern assembly are from the same part of the genome. However, a recent study^25^ has shown that the largest (12 Mb) of these repetitive scaffolds in the northern assembly is associated with the expansion of a novel transposable element and is not linked to any of the three previously identified divergent chromosome regions.

By fitting demographic models to the blockwise site frequency spectrum, we found that the northern and southern haplotypes have divergence times of ~1.2 Myrs across the three regions (Fig. 1). These estimates are considerably lower than the divergence time between the willow warbler and its closest relative, the chiffchaff, which is estimated to be around 5 Myrs^26^. Hence, we can reject the hypothesis that the presence of the divergent haplotypes within the willow warbler is a result of introgression from an extant *Phylloscopus* species. The divergence estimates are similar to those reported for large common inversion polymorphisms in several other study systems^3,4,7^, although more recent inversions would be harder to detect as they have accumulated less divergence.

Our analyses provide support for a previous hypothesis that the extant willow warbler is a result of an ancient hybridization event between two divergent populations^16^. The similar divergence times across the regions are compatible with a scenario where an ancestral population was split into two allopatric populations that subsequently hybridized with each other and homogenized the genome except for the divergent regions (Fig. 1). In this scenario, the estimated divergence times would represent the time around the population split, and the structural rearrangements would have appeared at some time between the population split and the secondary contact event. The inverted haplotypes could have segregated at low frequency in either of the allopatric populations and increased in frequency at the time of secondary contact due to positive selection^17^. In this case, the structural rearrangements would have been selected for because they protected favorable allele combinations in the divergent chromosome regions, for example, those associated with adaptations to specific migratory routes in each population, from being broken apart due to gene flow and recombination^27^.

The genome-wide changes in effective population size over time as determined from the MSMC2 analysis (Supplementary Fig. 6) are largely compatible with the proposed scenario of allopatric populations. The genome-wide effective population size could be increased when there is population structure^28^ and in willow warblers we observe an increase around the estimated divergence time of the northern and southern haplotypes. The decline in population size starting around 400 kya may then reflect the merging of the allopatric populations. However, the changes in genome-wide N_e_ could as well be caused by census population changes, although the scenarios are not mutually exclusive.

In the divergent regions on chromosomes 1 and 5, which are associated with differences in migratory phenotypes, we found evidence for rearrangements that are adjacent to or nested within each other (Fig. 2). Complex rearrangements have been observed in a wide range of taxa^7,29,30^ and are likely to reduce gene flow even further between populations. Alignments to the chiffchaff assembly and other bird assemblies enabled us to determine which of the subspecies has the derived or ancestral gene order within each region (Fig. 2). For the region on chromosome 1, the southern subspecies has the derived gene order, whereas in the region on chromosome 5, the northern willow warbler possess derived rearrangements. Unexpectedly, for the region on chromosome 3, the chiffchaff and the southern willow warbler both possess a derived rearrangement compared to the flycatcher and the zebra finch. The shared structural configuration between the northern willow warbler and the chiffchaff at the start of the region suggests that there have been two independent inversion events. Genomically unstable regions with recurrent inversions across species have previously been observed in mammals^31,32^.

The spread of the inverted haplotypes in either population at the secondary contact event could be expected to have given rise to a selective sweep that reduced variation^33^. However, we did not observe any consistent reduction in *N*_e_ for inverted haplotypes, which suggests that any sweeps happened sufficiently long ago for diversity to accumulate and/or that the inverted haplotypes had been segregating some time before the selection event took place and generated softer sweeps. Instead, the southern haplotypes were consistently assigned lower effective population sizes in the modeling analysis (Fig. 1) and had overall lower nucleotide diversity, higher Tajima’s D and a higher abundance of high-frequency derived alleles (Supplementary Figs. 7–9). The northern haplotypes are currently found over a larger geographical range than the southern haplotypes^13^, particularly for the chromosome 1 and 5 regions, and it is, therefore, plausible that they even historically have maintained larger effective population sizes.

Our analyses only favored an isolation with migration model for the region on chromosome 3, where there was migration from northern to southern populations (Fig. 1). The region on chromosome 3 markedly differs from the other two regions in its geographical distribution of northern and southern haplotypes^13,34^. While the divergent haplotypes for chromosomes 1 and 5 only meet at narrow migratory divides in Europe, the contact zone for the divergent haplotypes on chromosome 3 extends from central Scandinavia eastwards to southern Siberia, which likely allows for more opportunities for gene flow. Increased gene flow in this region may also be facilitated by the apparent lack of more complex rearrangements as seen in the other two regions (Fig. 2). Although not supported by simulations, we cannot rule out that there has been at least some gene flow between northern and southern populations also in the regions on chromosome 1 and 5. Double crossovers are, together with gene conversion, the main mechanism allowing for gene flow between inverted and collinear haplotypes, and are predicted to be less frequent closer to breakpoints^35^. Consistent with this prediction, we observed the highest differentiation in the vicinity of breakpoints in the regions on chromosomes 1 and 5 (Fig. 2).

We identified similar sequence repeats for at least some of the different breakpoints within each divergent region, which are likely to have been directly involved in the formation of the structural changes^36^. Interestingly, arrays of the same type of tandem repeat are associated with the breakpoint regions on chromosomes 1 and 3, and are found in both of the subspecies and in the chiffchaff, but not in the corresponding intervals in the zebra finch or collared flycatcher genome. Within the willow warbler genomes, highly similar and complete copies of this repeat (at least 50% length and 90% identity) are restricted to the two regions and on scaffolds predicted to be adjacent to them. The differentiated region on chromosome 5 in the southern assembly showed a different type of tandem repeat array at the start, as well as 31 kb segmental duplications containing a truncated and likely pseudogenized copy of the *URB1* gene at two of the breakpoints in the northern assembly.

Identifying selective targets within each divergent region is challenging due to the large number of genes (*N* = 47–197) and high linkage disequilibrium. Breakpoints themselves may be under selection if they modify the expression or disrupt the protein-coding sequence of genes^3,37^. However, none of the breakpoint intervals overlapped with or were very close to annotated functional genes (Supplementary Table 8), although we cannot rule out an effect on more long-distance regulatory elements.

We observed a clear reduction in diversity and an excess of high-frequency derived alleles in northern but not in southern willow warblers in an intron of the *SPON1* gene, which is located in the chromosome 5 region (Supplementary Fig. 10). This pattern is indicative of positive selection occurring in northern willow warblers and the sequence change may have a regulatory effect on the expression of the gene. *SPON1* has been shown to be important for axon guidance^38^ and has also been implicated in circadian rhythms^39^. Differences in this gene could, therefore, conceivably underlie some of the differences in migratory behavior observed between the subspecies. We also identified 73 highly differentiated SNPs or short indels in 46 genes that were predicted to modify the protein-coding sequence (Supplementary Table 9). These genes are associated with a wide range of biological processes and some of them lack any functional annotation. The variant with the largest predicted impact was a frameshift deletion in the *GTF3A* gene located on chromosome 1, which also contains three additional highly differentiated SNPs. This gene encodes a transcription factor involved in the transcription of 5S rRNA genes and has in humans been associated with body mass index^40^. The highly differentiated variants in this gene could potentially be associated with physiological adaptations to the different migratory routes of the subspecies. In line with this, we also found highly differentiated nonsynonymous mutations in three fatty acid desaturase genes that are located in tandem in the divergent region on chromosome 5. Fatty acid desaturase genes regulate the unsaturation of fatty acids and have been shown to underlie dietary adaptations in humans^41,42^.

The region on chromosome 3, on the other hand, shows a strong correlation with altitude and latitude in the breeding area^13,34^, and a potential selective benefit of the northern haplotypes could be increased cold tolerance. In this case, a potential candidate gene would be LDL receptor-related protein 11 (*LRP11*), which is annotated with the gene ontology term “response to cold”.

In order to identify additional putative functional differences, we also screened for highly differentiated structural variants. While most of these variants were located far away from the closest gene, a 207 bp insertion overlapped the three prime untranslated region of *STOML3* on chromosome 1 and could potentially have an effect on post-transcriptional regulation of this gene, which modulates the sensitivity of mechanoreceptors^43^. Mechanoreceptors are involved in several physiological processes^44^ and the potential phenotypic effect of the structural variant is, therefore, difficult to predict. It should, however, be noted that our ability to accurately genotype structural variants from the resequenced short-read samples is limited, particularly in more repetitive intervals, and future studies incorporating long-read data from additional samples are likely to uncover a broader spectrum of relevant structural differences.

Overall, the functional annotation of the nonsynonymous changes and the structural variants suggest that the regions affect several different gene pathways and could potentially have widespread phenotypic effects.

In conclusion, we have demonstrated that structural rearrangements maintain large differentiated regions despite extensive hybridization, and our results add to a growing body of evidence that structural rearrangements are often complex and associated with repeat expansions. Using a modeling approach, we obtained more robust estimates of divergence times and showed that the divergent regions of the three chromosomes are of similar ages. This observation is compatible with a scenario where the inversions arose in allopatric populations that later came into secondary contact and hybridized. Finally, our improved genome and annotation has provided a set of new candidate genes for adaptations related to migration and environmental gradients.

## Methods

The research in this study was performed in agreement with permission M45-14 issued by Malmö/Lund Ethical Committee for Animal Research, Sweden, which granted capture and blood sampling of wild birds

### Samples

Nine willow warblers, determined to be males (based on a wing length > 69 mm), were caught opportunistically with mist nets during the time of autumn migration in September 2016 at Krankesjön, 15 km East of Lund, Southern Sweden. While most of the individuals were phenotypically similar to willow warblers breeding in Southern Scandinavia, some were slightly larger and had a greyer plumage, which is more commonly seen in Northern Scandinavia^12^. The set of samples thus potentially contained willow warblers of each of the two major migratory phenotypes. Blood from each bird was collected through a puncture of the brachial vein and was stored in two vails containing SET buffer and 70% ethanol, respectively. An aliquot of the blood was used for DNA extraction with a phenol-chloroform protocol. From the extracted DNA, we genotyped the samples for two loci located on chromosomes 1 and 5, respectively (*NBEA* and *FADS2*)^45,46^, and for a bi-allelic marker within the divergent region on chromosome 3 (AFLP-ww1)^47^. Based on the genotyping results we selected two samples that were homozygous northern or homozygous southern for all three loci, respectively. We also included a sample from a chiffchaff *Phylloscopus collybita* (female) for de novo genome sequencing of a closely related outgroup species, as well as an additional willow warbler (DD81063, male) to confirm breakpoint differences with linked read sequencing. Both of these birds were opportunistically caught at the same site as above during autumn migration in 2019, and collection of blood followed the same approach as for the other birds.

### Optical maps

DNA from the northern and southern willow warbler was extracted from blood stored in ethanol using a Plug Lysis protocol (v.30026D; Bionano Genomics, CA, USA). The blood was first separated from the ethanol through gentle centrifugation and embedded in molten 2% agarose plugs (DNA plug kit; Bio-Rad, CA, USA). The solidified plugs were submerged in Lysis Buffer solution (Bionano Genomics) and 66.8 µl per ml Buffer Puregene Proteinase K (Qiagen,MD, USA) for 2 h at 50 °C. The plugs were subsequently washed in 1× Wash buffer (Bio-Rad DNA plug kit) followed by TE buffer. In the following step, the plugs were treated with RNase (Qiagen, 20 µl in 1 ml TE buffer) for 1 h at 37 °C, followed by another washing step using the same buffers as in the previous step. Next, the plugs were melted for 2 min at 70 °C and treated with GELase (Epicenter, WI, USA) for 45 min at 43 °C. The DNA was then purified from digested agarose using drop dialysis against TE buffer on a 0.1 µm dialysis membrane (MF-Millipore, Merck KGaA, Germany) for 2.5 h.

Optical maps for each of the two samples were produced using Bionano Genomic’s commercial Irys system^48^. BspQ1 was determined to be the most suitable nicking enzyme after using the software LabelDensityCalculator v.1.3.0 and Knickers v.1.5.5 to analyze a previous short-read assembly^13^. Bionano Genomic’s IrysPrep Labeling-NLRS protocol (v.30024) was used for the NLRS reaction. For this step, DNA was treated with Nt.BspQ1 (NEB, MA, USA) to create single-stranded nicks in a molecule-specific pattern. These were then labeled with Bionano Genomic’s (CA, USA) labeling mix (NLRS kit), aided by Taq Polymerase (NEB), and repaired using Bionano Genomics’s repair mix (NLRS kit), in the presence of Thermopol Rxn buffer, NAD+, and Taq DNA Ligase (NEB). Finally, the DNA backbone was stained using DNA stain from Bionano Genomics’s NLRS kit. Each sample was then loaded on two IrysChips (Bionano Genomics) each, and the DNA with stained BspQ1 nicks was visualized using an Irys instrument, following Bionano Genomics’s Irys user guide (v.30047). This resulted in 200 and 182 Gb of data for the northern and southern sample, respectively.

Genome maps were assembled de novo using Bionano Genomic’s in house software IrysView v.2.5.1, with noise parameter set to “autonoise” and using a human arguments xml file. The genome map was then further refined by re-assembling all data, but using the first assembly version as a reference. The final assemblies were both 1.3 Gb in total size, with an average coverage of 92.3 and 96.4×, and N50 of 0.93 and 0.95 Mb, for the northern and southern sample, respectively.

### Linked read sequencing

For the southern sample and sample DD81063, DNA for chromium sequencing (10× Genomics, CA, USA) was extracted from blood stored in SET buffer using a MagAttract HMW DNAkit (Qiagen) at Scilifelab, Stockholm, Sweden. For the northern sample the extraction for bionano optical maps was used. The libraries of the northern and southern sample were each sequenced on a separate lane of a HiSeqX (Illumina, CA, USA) and the DD81063 sample was sequenced on a NovaSeq6000 (Illumina). For all samples sequencing was performed using a 2 × 150 bp setup.

### Northern willow warbler de novo assembly

Library preparation for long read sequencing was done on DNA previously extracted for the optical map and followed Pacific Bioscience’s (CA, USA) standard protocol for 10–20 kb libraries. No shearing was performed prior to the library construction, but the library was size selected using the BluePippin pulse field size selection system (Sage Science, MA, USA), with a size cut-off >25 kb. The library was sequenced on eight SMRT cells on a Sequel platform (Pacific Biosciences). The sequencing yielded 63.66 Gbp of data comprised of 4,690,365 subreads with a mean length of 13,573 bp (range: 50–170,531 bp).

The Pacbio reads were assembled de novo in HGAP4^49^ in the SMRT Link package with default settings except for specifying an expected genome size of 1.2 Gbp and setting the polishing algorithm to “Arrow”. We ran Falcon unzip^50^ on the assembly to obtain partially phased primary contigs and fully phased haplotigs. Within the software, Arrow was used to polish the assembly using reads assigned to each haplotype. We evaluated two unzipped assemblies based on 30× or 40× coverage of seed reads in the preassembly step in HGAP4. A lower coverage threshold will lead to longer reads in the initial assembly step, which may increase the contiguity of the assembly, but will on the other hand, limit the number of reads that can be used in the phasing and polishing step. Although the unzipped assemblies were very similar, the 40× version was chosen for downstream analyses as it was slightly more contiguous and contained a higher number of single-copy bird orthologues as determined by BUSCO version 3.0.2^51^.

The assembly was further polished with Pilon 1.22^52^ with Illumina chromium reads from the same sample. The Illumina reads were mapped to the assembly using bwa version 0.7.17-r1188^53^ and duplicated reads were marked using picardtools 2.10.3 (http://broadinstitute.github.io/picard). Pilon was run by only correcting indels and in total the software made 1,043,827 insertions and 275,457 deletions, respectively, of which the vast majority (94%) were single basepair changes. The Illumina polishing had a pronounced effect on the number of single-copy bird orthologues that could be detected in the primary contigs (Supplementary Table 1).

For further assembly steps, we extracted the Illumina-polished primary Pacbio contigs (*N* = 2737, N50 of 2.1 Mb and a length of 1.29 Gb). These contigs showed an unexpectedly high level of duplicated single-copy orthologues (7.4%), which suggested partial or complete overlap between some contigs. As a first step to reduce the redundancy and increase the contiguity of the assembly, we hybridized the primary contigs to the optical map of the same sample using bionano solve version 3.2.2 (BioNano Genomics) with default settings except for specifying aggressive scaffolding parameters. The hybrid scaffolding resulted in 19 cuts to the bionano maps and 259 cuts to the Pacbio contigs and created 363 super-scaffolds. Most of the gaps between the contigs in the super-scaffolds were estimated to be negative (i.e., some overlap between sequences). However, in the hybrid assembly, sequences on either side of these gaps were not collapsed and thus formed false segmental duplications. To remedy this problem we extracted 304 sets of overlapping contigs (“supercontigs”) and used GAP5 in the staden package 2.0.0.b11^54^ to find potential joins between the contig ends. Using this approach, we merged contigs at 558 (87%) of the putative overlaps. The mean alignment length in the overlaps was 111 kb (range: 0.259–661 kb) with a mean sequence divergence of 3.28% (range: 0.31–15.55%). The highest divergence was caused by the presence of large indels. By trimming off one or both ends of the contigs at the gaps (mean 23 kb, range: 0.6–60 kb), we were able to close 23 further gaps. For the remainder of gaps, GAP5 failed to find potential joins between contigs or the ends supposed to be joined were considered to have too high divergence. The new assembly, including supercontigs consisted of 2401 contigs with an N50 of 6.5 Mb and had a considerably lower amount of duplicated single-copy genes (4.6% vs 7.4%).

To further reduce the redundancy, we used the purge haplotig pipeline^55^ (downloaded 2019-02-15) to remove contigs that could be mapped over most of their length to larger contigs and that showed limited diploid coverage. We first estimated coverage by mapping the Pacbio subreads used for the de novo assembly with minimap2 version2.13-r860^56^ using default settings for Pacbio reads (-x map-pb). To minimize the loss of repetitive sequences that could be separated and scaffolded by the bionano optical map, we used the first bionano hybrid assembly (363 superscaffolds and 1500 cut and unscaffolded contigs) as a reference for mapping. From the mapped data we detected a clear haploid and diploid peak and set a threshold of diploid coverage above 34× and below 85×. Any scaffold where less than 80% of its positions had diploid coverage was considered a putative haplotig and was mapped to other scaffolds using minimap2 within the software. We removed 1209 scaffolds (mean size: 107,655 bp, range: 598–495,788 bp) with a coverage to the best hit of at least 70% (mean: 97.4%). Using this approach, we specifically excluded contigs that could not be incorporated in superscaffolds. However, we also removed three contigs that each entirely made up short superscaffolds that could be uniquely assigned to larger superscaffolds and that had a high degree of haploid coverage. At this stage, we also removed five additional contigs shorter than 1000 bp that were the result of cutting the assembly with the bionano optical map. This led to an assembly with 1187 contigs, a length of 1.1 Gbp and a N50 of 7.9 Mb. The filtered assembly showed a large reduction in single-copy orthologue bird genes (1.3 vs 4.6%).

To provide an intermediate level of scaffolding to the optical map, we mapped the 10× chromium reads of the same sample to the assembly using bwa and used arcs version 1.0.5^57^ and LINKS version 1.8.6^58^ for scaffolding. Arcs was run with default settings except for enabling gap size estimation (--dist_est) and LINKS was run by setting the number of supporting links to at least 5 (-l = 5) and the maximum link ratio between the two best contig pairs to 0.3 (-a = 0.3). The scaffolding resulted in 739 scaffolds with a N50 of 16.4 Mb and a length 1.12 Gb.

As a final scaffolding step, we hybridized the 10× chromium-Pacbio scaffolds to the bionano optical map using the same settings as before. The hybrid scaffolding made 23 cuts to the optical map, 122 cuts to the scaffolds and resulted in 497 scaffolds with an N50 of 16.8 Mb. Two contigs representing the divergent region on chromosome 1 had been scaffolded together by arcs but were separated and not re-scaffolded with other sequences in the bionano hybrid assembly. Since the mismatched end of the optical map was short, located at a large gap, and the gene order is the same as seen in other bird genomes, we decided to keep the scaffold generated by arcs.

For this round of hybrid scaffolding, there were 52 gaps that were estimated to be negative. Using the same approach as when creating supercontigs, we were able to close 10 of these gaps. We additionally closed gaps using PBJelly^59^ from PBSuite 15.8.24 with default settings except for specifying --spanOnly --capturedOnly”. The software filled 97 gaps, extended one end of 12 gaps, extended both ends of 18 gaps and overfilled 28 gaps (extended both ends but detected no overlap despite the extension is larger than the predicted gap).

We further checked for potential misjoins between scaffolds that originate from different chromosomes. To this end, we used SatsumaSynteny 2.0^60^ to produce whole-genome alignments between the assembly and the genomes of chicken (version GRCg6a) and zebra finch (version taeGut3.2.4), both downloaded from Ensembl (www.ensembl.org). Using this approach, we detected a scaffold that showed good alignments to both chromosomes 10 and 23 in both of the other species. We considered this join unlikely and decided to split the scaffold.

Next, we performed a second round of polishing with the 10× chromium Illumina data from the same sample. For this round, since we had fewer than 500 scaffolds, we used the longranger 2.1.14 align pipeline^61^ to map reads in a barcode-aware way. Pilon was then run with the same settings as before and resulted in the correction of 417,032 indels, of which 78.7% were single-basepair changes. The second round of polishing considerably increased the number of single-copy bird orthologues that could be identified in the assembly (Supplementary Table 1).

The mitochondrial genome was not found in the original Pacbio genome assembly. We obtained this genome by adding the complete mitochondrial sequence from a previous short-read assembly^13^. We then used bwa to map the 10× chromium reads from the northern sample to the assembly and extracted alignments on the mitochondrial sequence. Next, freebayes was used with a haploid setting to detect differences present in the aligned reads. The raw variant file was filtered with vcftools for sites with a quality less than 30 and for two intervals with excessive read coverage (possibly reads from unassembled NUMTs). The filtered variant file contained 11 substitutions and three indels, and was used with bcftools version 1.14^62^ to create a new mitochondrial reference.

For the extraction and removal of sequences in the different assembly steps we used kentUtils 370 (https://github.com/ucscGenomeBrowser/kent). Summary statistics for each assembly (e.g., N50) were calculated using the assemblathon_stats.pl script^63^.

### Southern willow warbler and chiffchaff de novo assemblies

The southern willow warbler and the chiffchaff were each sequenced on two lanes on a Sequel II (Pacific Biosciences) using a high-fidelity (HiFi) setup. Sequencing libraries for the southern willow warbler was prepared from a previous extraction used for optical maps (see above), whereas for the chiffchaff, DNA was extracted from blood using a Nanobind extraction kit (Circulomics, MD, USA). The southern willow sample yielded 2,576,876 HiFi reads with a mean length 19,303 bp and representing 49.7 Gbp. The chiffchaff sample yielded 2,612,165 HiFi reads with a mean length of 19,829 bp and representing 51.8 Gbp.

The HiFi reads were assembled de novo using hifiasm version 0.15.5-r350^64^ with default settings and primary contigs were selected for downstream analyses. For the chiffchaff hifiasm assembly, we removed the first 6 Mb part of a contig overlapping with another contig and removed a short interval at the end of a contig containing adaptor sequences. For the southern willow warbler, the primary contigs (*N* = 540, Supplementary Table 1) were hybridized to the optical map of the same sample using the same pipeline as for the northern sample. Although we had access to chromium data from the same sample, we did not include it to perform an intermediate scaffolding step (as we did for the northern willow warbler assembly) because the long-read assembly was already highly contiguous. The hybridization step made 39 cuts to the contigs and 20 cuts to the optical maps, resulting in an assembly with 111 superscaffolds and 439 non-scaffolded contigs. We decided to ignore an optical map-supported fusion of contigs that mapped to separate chromosomes in other bird species, as this fusion was made in a large repetitive region. We further excluded a 45 bp sequence resulting from the hybrid assembly cutting and masked four short intervals containing adaptor sequences. The assembly of the mitochondrion in the southern assembly followed the same pipeline as used for the northern assembly (see above). In this case, 10 substitutions and two indels were added to the mitochondrial sequence from the previous short-read assembly based on alignments of linked reads from the southern sample.

### Repeat annotation

We used Repeatmodeler version 1.0.8^65^ for de novo identification of repeats in the southern assembly. The repeats detected by repeatmodeler were combined with 1,023 bird-specific repeats into a custom library. Next, we used repeatmasker version 4.0.7^66^ with the custom library and by using a more sensitive search (-s flag) to annotate repeats in the genome. Bedtools v2.29.2^67^, together with the annotated repeats, was used to create a softmasked version of the southern assembly, which was used in the gene annotation step. The same repeat library was also used to annotate repeats in the de novo assembly of the northern sample. For the chiffchaff assembly we used the same annotation approach as for the southern willow warbler, but included a species-specific library generated with repeatmodeler, and also included a tandem-repeat associated sequence associated with the divergent regions on chromosomes 1 and 3 from the willow warbler library. Intervals with tandem repeats in divergent regions were also analyzed with tandem repeats finder version 4.0.9^68^ using default settings except for specifying a maximum period size of 2000 bp.

Duplicated intervals within divergent scaffolds were identified with Minimap2 and subsequently aligned with EMBOSS Stretcher 6.6.0 (https://www.ebi.ac.uk/Tools/psa/emboss_stretcher/).

### RNA sequencing

We used total RNA extracted from whole brain from six samples used in an earlier study quantifying differential expression in migratory and breeding willow warblers^69^ (Supplementary Table 3). The quality of the RNA was checked with a Bioanalyzer version 2100 (Agilent, CA, USA). All of the extractions had a RNA Integrity Number (RIN) of at least > 7.10. RNA libraries for sequencing were prepared using a TruSeq Stranded mRNA Sample prep kit with 96 dual indexes (Illumina) according to the instructions of the manufacturer with the exception of automating the protocols using an NGS workstation (Agilent) and using purification steps as described in Lundin et al^70^. and Borgström et al^71^. The raw RNA data was trimmed using cutadapt version 1.8^72^ within Trim Galore version 0.4.0 (https://github.com/FelixKrueger/TrimGalore) with default settings.

We used Stringtie version 1.3.3^73^ to create transcripts from the RNAseq data. These transcripts were not used directly in the generation of gene models, but used in the manual curation step as potential alternative transcripts. For the software, we first mapped the reads with Hisat2 version 2.1.0^74^ using default settings for stranded sequence libraries and downstream transcript analyses.

### Gene annotation

We used Augustus version 3.2.3^75^ to create gene models using hints provided from RNAseq data and protein data from other bird species. For the RNAseq data, we mapped the trimmed reads to the assembly using STAR version 2.7.9a^76^. Accessory scripts in the Augustus package were used to filter the alignments for paired and uniquely mapped reads and for extracting intron hints. We additionally generated coverage wig files for each strand from the filtered alignment file using the software stranded-coverage (https://github.com/pmenzel/stranded-coverage) and used these as input for the august wig2hints.pl to generate exonpart hints.

For homology evidence, we downloaded a set of bird proteins from NCBI (https://www.ncbi.nlm.nih.gov/). This data set included 49,673 proteins from chicken, 41,214 proteins from zebra finch and 38,619 proteins from great tit. We also downloaded an additional dataset from Uniprot (www.uniprot.org) that consisted of 3175 manually reviewed bird proteins and 204 and 12,263 bird proteins that were not manually reviewed but supported by protein or transcript data, respectively. The protein data was mapped to the genome using exonerate version 2.4.0^77^. We used the script align2hints.pl from braker 2.1.6^78^ to generate CDSpart, intron, start and stop hints from the data.

Augustus was run with species-specific parameters (see training Augustus below) and with default settings except for specifying “softmasking=true”, “--alternatives-from-evidence=true”, “--UTR = on”, “--gff3=on” and “--allow_hinted_splicesites=atac”. In the extrinsic configuration file, we changed the malus for introns from 0.34 to 0.001, which increases the penalty for predicted introns that are not supported by the extrinsic data (RNAseq and protein hints). The prediction resulted in 28,491 genes and 35,389 transcripts.

The Augustus-derived gene models were assigned names based on overlap with synteny-transferred zebra finch genes. For this purpose, we used SatsumaSynteny with default settings to obtain whole-genome alignments between our assembly and the zebra finch genome version bTaeGut1.4.pri^79^. Based on the alignment, we used kraken^80^ (downloaded 2020-04-14) to transfer the zebra finch genome annotations (NCBI Release 106) to the willow warbler assembly. We then extracted the CDS from the Augustus gene models and the kraken genes and used bedtools intersect to quantify the overlap. The gene models were also searched against the longest translation of each of the chicken, zebra finch and great tit *Parus major* genes used as evidence for the gene prediction step and against 86,131 swissprot vertebrate proteins using blastp 2.5.0+^81^ with an E value threshold of 1e−5. Gene models that were not annotated through synteny were assigned a gene name based on the blast results. Protein domains in the gene models were annotated with interproscan v 5.30–69.0^82^. To reduce the number of false positive predictions we removed 5697 genes that were not supported by synteny to zebra finch genes, showed no significant similarity to vertebrate proteins or did not contain any annotated protein domains.

We used Webapollo 2.6.5^83^ to manually curate gene models in the previously identified divergent chromosome regions and in other regions where differences were present. In the curation step, we specifically validated the support for the coding sequence and the UTR and also removed genes that were likely to be pseudogenes based on a truncated coding sequence compared to homologous genes in other vertebrates, had no support from synteny in other bird species and/or that were located in repeat-rich regions.

### Training Augustus

We used a previous repeat-masked short-read assembly^13^ and the trimmed RNAseq data used in this study to obtain species-specific parameters for Augustus. The RNAseq data was assembled into transcripts using Trinity version 2.0.2^84^ to create a de novo and a genome-guided assembly that together were comprised of 1,929,396 transcripts. The genome-guided transcript assembly was based on RNAseq mapped to the genome using GSNAP version 2016-07-11^85^ with default settings. We used PASA version 2.0.2^86^ to create high-quality transcripts, which were imported into Webapollo. To assess the completeness of the transcripts, we compared them to synteny-transferred models from the chicken genome using Kraken. We selected 1249 transcripts that appeared complete, were not overlapping with other genes and showed less than 80% amino acid similarity to another gene in the training set. From this set, we excluded 21 genes that were giving initial training errors, which gave us a training set of 1228 genes. This gene set was randomly split into 1028 training genes and 200 genes used for testing. For training, we used the optimize_augustus.pl script with default settings except for the flag –UTR = on.

### Whole-genome resequencing and variant calling

We used the whole-genome resequencing data from nine samples of each migratory phenotype provided in Lundberg et al^13^. and sequenced an additional two high-coverage samples from each migratory phenotype (Supplementary Table 4). Sequencing libraries for the new samples were prepared with a TruSeq DNA PCR-Free kit (Illumina) with a targeted insert size of 670 bp or with a Truseq DNA nano (Illumina) with a targeted insert size of 350 bp. All of the new samples were sequenced on a HiSeqX (Illumina). The raw reads were trimmed with trimmomatic 0.36^87^ with the parameters “ILLUMINACLIP:TruSeq3-PE-2.fa:2:30:10 LEADING:3 TRAILING:3 SLIDINGWINDOW:4:15 MINLEN:30”.

Quality-trimmed reads were mapped to the southern assembly using bwa mem with default settings except for specifying -M flag to ensure compatibility with the downstream duplicate removal steps and converted into binary alignment map (bam) files using samtools. For samples sequenced across multiple lanes, reads from each lane were mapped independently and the resulting bam files were merged with samtools. Read duplicates were removed with the markduplicates tool provided in picardtools.

From the aligned whole-genome resequencing data set, we called variants with freebayes v1.1.0 using default settings and parallelizing the analyses of separate scaffolds using GNU parallel^88^. Vcflib version 2017-04-04^89^ was used to filter the raw set of variants for sites with quality score >30 and for alternate alleles that were supported by at least one read on each strand (SAF > 0 & SAR > 0) and had at least one read balanced to the right and the left (RPL > 0 & RPR > 0). Next, we used vcftools 0.1.16^90^ to filter genotypes with a coverage of at least 5x and removed sites a maximum of four genotypes missing in each of the populations. The variants were also filtered for collapsed repeats by removing sites with a mean coverage of more than twice the median mean coverage (30×). We next used vcflib to decompose haplotype calls and complex alleles into indels and SNPs and removed any variants that were overlapping with annotated repeats. This gave us a final of 51 million variants of which 45 million were bi-allelic SNPs. We used vcftools to calculate F_ST_^91^ for each variant and for bi-allelic SNPs in non-overlapping windows of 10 kb. As many rare variants segregate in the willow warbler populations, which may downwardly bias differentiation estimates^92^, we focused on variants with a minor allele frequency of at least 0.1.

Coverage for each resequenced sample was calculated in non-overlapping 1 kb windows using bedtools and only included properly paired reads with a mapping quality of at least 1. The raw coverage values for each sample were normalized by its median coverage across all windows.

### Structural variant calling

We used a combination of delly 0.9.1^93^ and GraphTyper 2.7.4^94^ to call structural variants in the resequenced samples. To identify a set of high confidence variants, we first mapped the long reads from the northern willow warbler to the southern assembly using minimap 2.22-r1101^56^ with default settings for Pacbio reads and from the alignments called variants using delly. Next, GraphTyper was used to genotype the resequenced samples for the delly variants in the scaffolds containing the divergent chromosome regions. The raw set of variants were filtered to contain only sites with a “PASS” flag and, for each variant, the aggregated genotype, which is the genotype model out of breakpoint alignments and coverage that has the highest genotyping quality, was chosen for downstream analyses. Genetic differentiation (F_ST_) was calculated in vcftools and variants with F_ST_ ≥ 0.7 between homozygotes in each divergent chromosome region were extracted and checked for overlap with genes and gene features using bedtools. To get more reliable differentiation estimates, we only included sites where at least 80% of the southern and northern homozygotes had genotypes.

### Inversion genotypes for resequenced samples

The resequenced samples were assigned a genotype of southern and northern haplotypes for each of the divergent regions based on a multidimensional scaling (MDS)-based clustering in invclust^95^ of SNP array genotypes in Lundberg et al.^13^. To obtain genotypes of the SNPs included on the array in the resequenced samples, we mapped the SNP array probe sequences to the northern assembly using gmap and from the alignments extracted the positions of the focal SNPs. Next, we used freebayes to genotype the resequenced samples for these positions and plink version 1.9^96^ to combine the genotypes with the genotypes from the SNP array. In the genotyping step, we also included mapped 10× chromium libraries for the northern and southern reference samples and the additional willow warbler sample. From the combined dataset, we extracted genotypes for SNPs located in each of the divergent regions and used invclust to assign each sample a genotype of inverted and non-inverted haplotypes. The inverted and non-inverted haplotypes were recoded as southern or northern haplotypes based on their frequency in each subspecies.

### Breakpoint analyses

We used MUMmer 4.0.0rc1^97^ to align the genomes of the southern and northern willow warblers, and the southern willow warbler genome to the genomes of the chiffchaff, zebra finch (3.2.4) and collared flycatcher FicAlb (1.5)^98^.

To provide further evidence of breakpoints, we mapped the 10× chromium reads of each sample to both the northern and the southern assembly and called structural variants using the longranger wgs pipeline. For the southern genome, we selected the 499 largest scaffolds and concatenated the rest into a single scaffold to make it compatible with the software. We also checked for differences in linked read molecule coverage between the samples. For this purpose, the raw reads of each sample were first processed with longranger basic for quality trimming and barcode processing. The trimmed reads were mapped to the assemblies using bwa mem using a -C flag to extract the barcode information of each read and alignments converted into bam files using samtools. To estimate coverage of barcodes, we first used the tigmint-molecule script from tigmint 1.1.2^99^ to obtain positional information of barcodes (molecules) in each divergent region. The software was run with default settings except for only using reads with a mapping quality of at least 1 and only to report molecules that were estimated to be at least 10 kb. We next used bedtools to count the number of overlapping molecules in 1 kb windows.

We explored differences between optical maps by using the runSV.py script in bionano solve with the southern optical map as a query and the northern assembly as target and the reciprocal analysis with the northern optical map as a query and the southern assembly as a target. We also used the bionano solve hybrid assembly pipeline to visualize differences between the optical maps and the genome assemblies at breakpoint regions.

### Functional annotation of differences

We used bedtools to quantify the distance between breakpoint intervals and annotated genes. To provide a functional annotation of the SNPs and short indels, we selected variants that showed a F_ST_ ≥ 0.7 between southern and northern homozygotes for each of the region and used these as input to Snpeff 5.0.0e^100^ together with the annotation and reference genome. We used Snpsift 5.0.0e^101^ to select variants that were predicted to have a moderate to high effect on genes. Gene ontology terms for the genes were extracted from orthologous genes in other bird genomes in ensembl (www.ensembl.org) or through domain searches of the proteins with interproscan.

### Age estimation and demographic analyses of divergent regions

In order to estimate the timing of the inversion events, we used high-coverage resequencing data from two southern samples, two northern samples and, as an outgroup, one dusky warbler *Phylloscopus fuscatus* (Supplementary Table 4). The willow warbler samples were chosen so that they were either homozygous southern or northern for all of three divergent regions. The dusky warbler library was prepared using a TruSeq Nano DNA library prep kit for Neoprep (Illumina) according to the instructions of the manufacturer and sequenced on a HiSeq X (Illumina). Quality-trimming of the raw reads and mapping of the trimmed reads to the northern reference genome followed the same approach as used for the willow warbler resequencing samples (see above).

Variants were called using freebayes and the raw set of variants were filtered using gIMble’s preprocess module (v0.6.0). Sample-specific callable sites were identified using gIMble preprocess and were defined as those with a minimum coverage of 8× and a maximum of 0.75 standard deviations above the mean coverage. Genic and repetitive regions of the genome were removed from the callable sites in order to limit downstream analyses to intergenic regions.

Summary statistics of genetic variation (π and *d*_*xy*_) within the divergent regions were calculated using gIMble. Following this, net divergence (*d*_a_) between northern and southern samples was calculated as *d*_north–__south_ − (π_north_ + π_south_)/2. To convert the net divergence into years we used the germline mutation rate (4.6 × 10^−9^) estimated in the collared flycatcher^21^. Relative node depth (RND) using the dusky warbler (DW) as an outgroup was calculated as *d*_north–south_/(*d*_DW-north_ + d_DW-south_)/2. For each divergent region, a blockwise site frequency spectrum (bSFS) was generated with gIMble using blocks of 64 bp in length. This length refers to the number of callable sites within a block, while the physical length of blocks was allowed to vary due to missing data but was limited to 128 bp. Downstream analyses that relied on a bSFS used a kmax of 2, meaning that only marginal probabilities were calculated for mutation counts >2. The composite likelihood (CL) of a model, given the bSFS of one of the divergent regions, was optimized using the Nelder-Mead algorithm with the maximum number of iterations set to 1000. Within the software we evaluated three different population models. The first model was a strict isolation model (SI), with parameters ancestral effective population size, effective population sizes for southern and northern willow warblers and divergence time. The second model was an isolation with migration model (IM_1_) that also included a migration rate from northern to southern samples, and the third model (IM_2_) instead had a migration rate from southern to northern willow warblers.

Simulations were carried out by msprime 0.7.4^102^ through gIMble. The recombination rates used for these simulations were chromosome-specific estimates from a high-density recombination map of the collared flycatcher^98^ and were 2.04, 1.95, and 2.63 cM/Mb for chromosomes 1, 3, and 5, respectively. A total of 100 replicates were simulated for the optimized SI parameters of each region. These simulated bSFSs were then optimized under both an SI model as well as the best fitting IM model for that region. The improvement in CL between these models was used as a null distribution for testing whether improvements in CL observed for the real data were greater than expected given a history of no migration. For each parameter, we calculated 95% CI as Maximum Composite Likelihood (MCL) estimate ± 1.96 * standard deviation of simulations (Supplementary Table 7). As a result, our estimates of uncertainty are affected by the recombination rates that we assumed for simulations. We also used the results of simulations to quantify the potential bias in MCL estimates due to intra-block recombination (Supplementary Table 7). However, we did not attempt to correct for this bias as it is relatively small (e.g., the MCL divergence times are estimated to be biased upwards by 7, 24, and 10%) and our estimation of the bias itself is largely dependent on the recombination rates we assumed.

MSMC2^24^ was used to explore genome-wide changes in N_e_ through time. As input to the software, we used the callable intergenic bed file and filtered vcf file mentioned above, with the addition of further filtering the bed file to only include autosomal scaffolds ≥500 kb and excluding the divergent regions. The input files for MSMC2, i.e., an unphased set of heterozygous sites for each sample, were generated using the generate_multihetsep.py script from msmc-tools. MSMC2 was run with a starting ρ/μ of 1 for 30 expectation-maximum iterations. For both the demographic modeling and MSMC2, we used the collared flycatcher germline mutation rate^21^ and a generation time of 1.7 years^11^ to convert divergence times into years.

To infer the effects of demographic events and selection, we also calculated several genetic summary statistics. To this end, we first imputed missing genotypes and inferred haplotypes for the filtered set of variants using beagle version 5.4^103^. From the full set of samples, we selected 10 and seven samples that were homozygous southern or northern for the three divergent regions, respectively, as determined from the MDS analysis (see above), and extracted bi-allelic SNPs. To identify ancestral and derived alleles, we extracted genotypes for the focal SNP positions from the aligned chiffchaff and dusky warblers reads using bcftools 1.14^62^ with the mpileup command. As a conservative approach, we considered any site with the presence of both the reference and alternate allele as heterozygous (regardless of their frequencies) and only included sites where the coverage was at least one-third of the mean coverage among all sites for each outgroup species. We next used a customized script to extract the sites from the original vcf files, and, if necessary, switch the reference and alternate allele and swap the genotypes accordingly. With the polarized genotype data, we used PopGenome 2.7.5^104^ to calculate Fay and Wu’s H and vcftools to get counts for the derived allele. We further used selscan 1.3.0^105^ to calculate XP-nsl^106^ between the southern and northern samples, Sweepfinder2^107^ to calculate a composite likelihood ratio (CLR) between a model where a selective sweep has had an effect on the allele frequency and a model based on the genome-wide allele frequency spectrum and used vcftools to calculate nucleotide diversity, Tajima’s D and linkage disequilibrium (D’).

The use of the southern assembly as a reference could potentially lead to a mapping bias for reads from southern samples, particularly in regions of higher divergence between the subspecies. This, in turn, could have an effect on genetic summary statistics and demographic modeling estimates. To explore the effect of reference bias, we therefore also mapped the resequencing data to the northern assembly, performed variant calling and calculated nucleotide diversity and Tajima’s D in 10 kb windows. For the northern assembly, we also used the same demographic modeling as used for the southern assembly. Contrasting average genetic summary statistics and demographic parameter estimates, we found negligible differences between the two genome assemblies (Supplementary Table 10).

### Reporting summary

Further information on research design is available in the Nature Portfolio Reporting Summary linked to this article.

## Supplementary information

Supplementary Information

Reporting Summary

## Data Availability

Raw sequence data, optical maps and de novo assemblies generated in this study are available at NCBI under bioproject PRJNA550489. Whole-genome resequencing data used from a previous study are available at NCBI under bioproject PRJNA319295. Figure source data and annotation files are available at Figshare (10.6084/m9.figshare.21821328.v1).

## Code availability

Workflows and scripts are available at Github (https://github.com/maxlundberg/warbler_inversions)^108^.
