# Supplementary material for: Inversions maintain differences between migratory phenotypes of a songbird
Source: Nat Commun. 2023 Jan 27;14:452. doi: 10.1038/s41467-023-36167-y (PMC9883250; doi:10.1038/s41467-023-36167-y)
Supplement: Supplementary file 1 — Supplementary Information [file 41467_2023_36167_MOESM1_ESM.pdf]

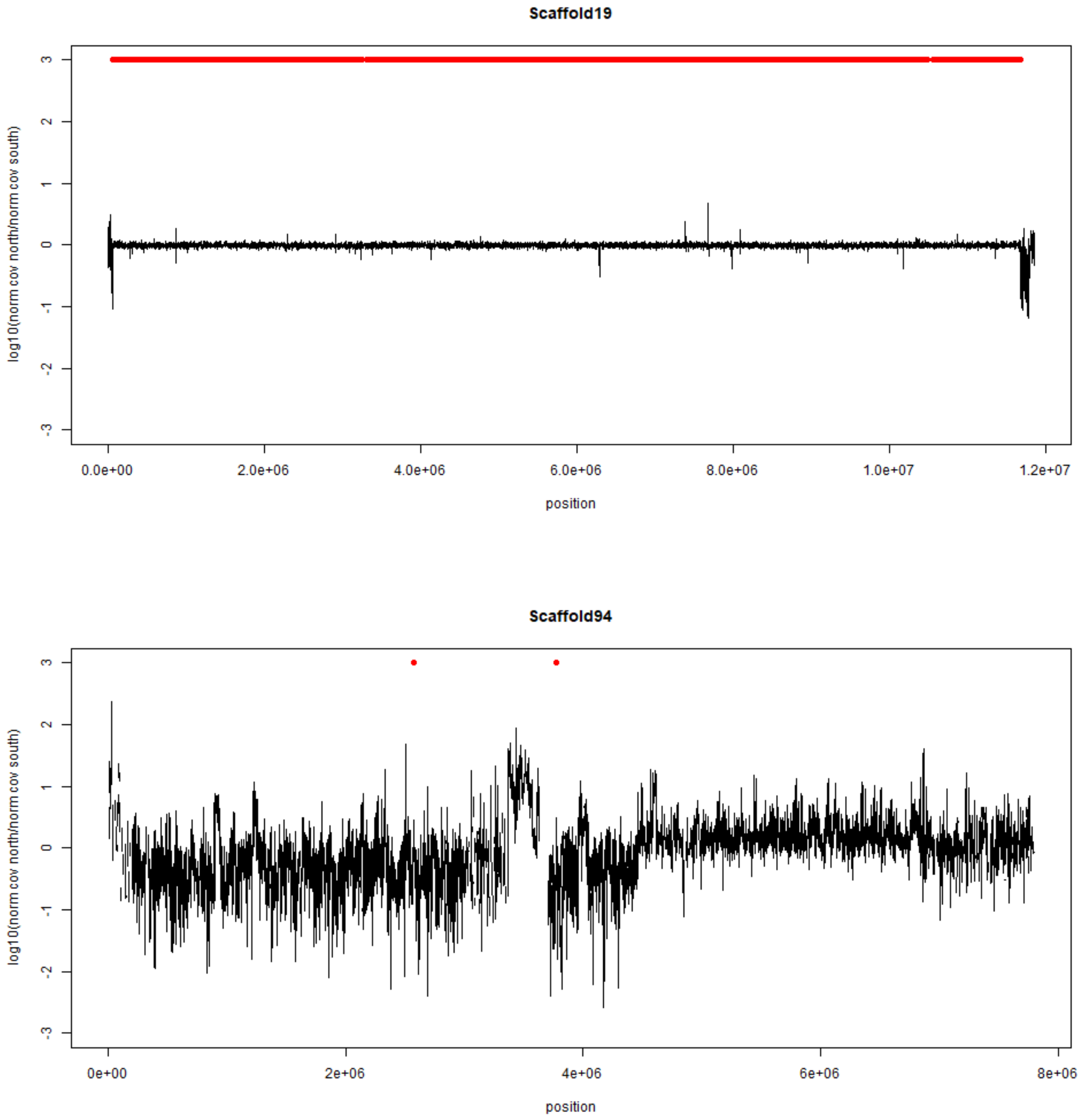

Supplementary Figure 1. Difference in mean normalized coverage ($\log_{10}$(norm cov_{north}/norm cov_{south})) between 11 northern and 11 southern resequenced willow warblers for two scaffolds in the southern assembly. Scaffold19 contains the divergent region on chromosome 1. Scaffold94 is a repeat-rich scaffold that contains highly differentiated variants between the willow warbler subspecies, but it has not been assigned to a specific chromosome. Coverage has been calculated in non-overlapping windows of 1 kb and only for properly paired reads with a minimum mapping quality of 1. Red points refer to positions with highly differentiated ($F_{ST}\geq 0.7$) variants.

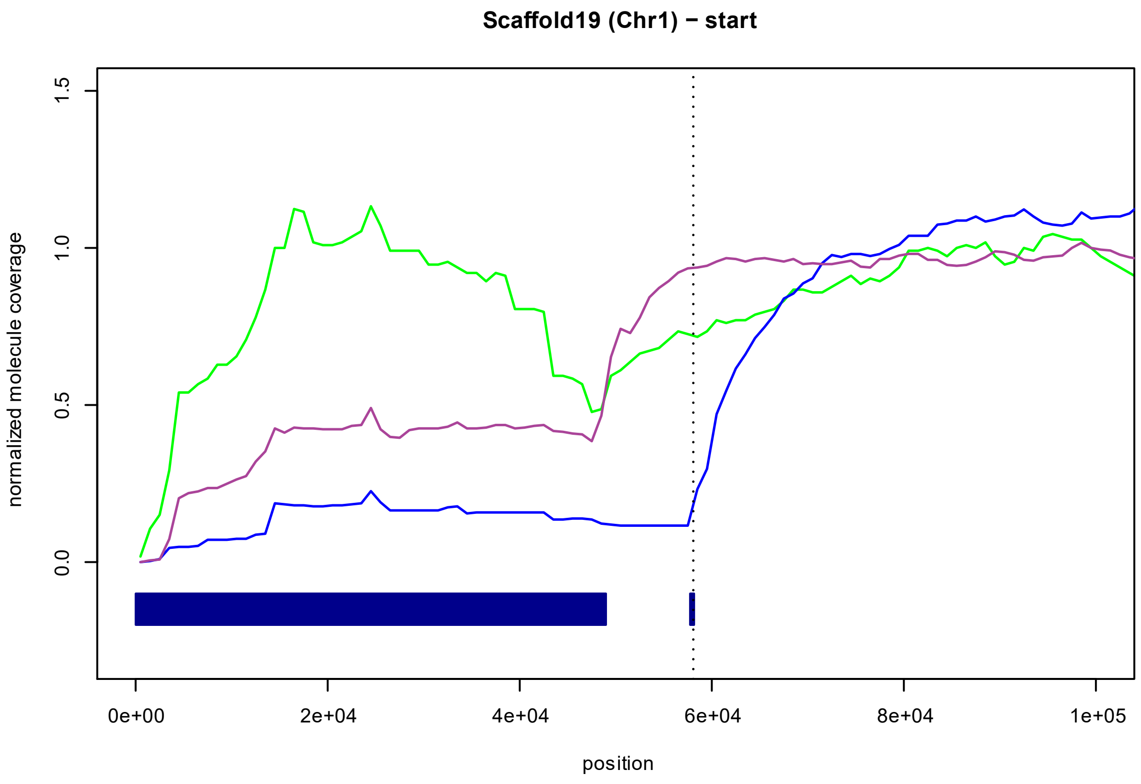
Scaffold19 (Chr1) − start
normalized molecule coverage
0.0
0.5
1.0
1.5
0e+00
2e+04
4e+04
6e+04
8e+04
1e+05
position

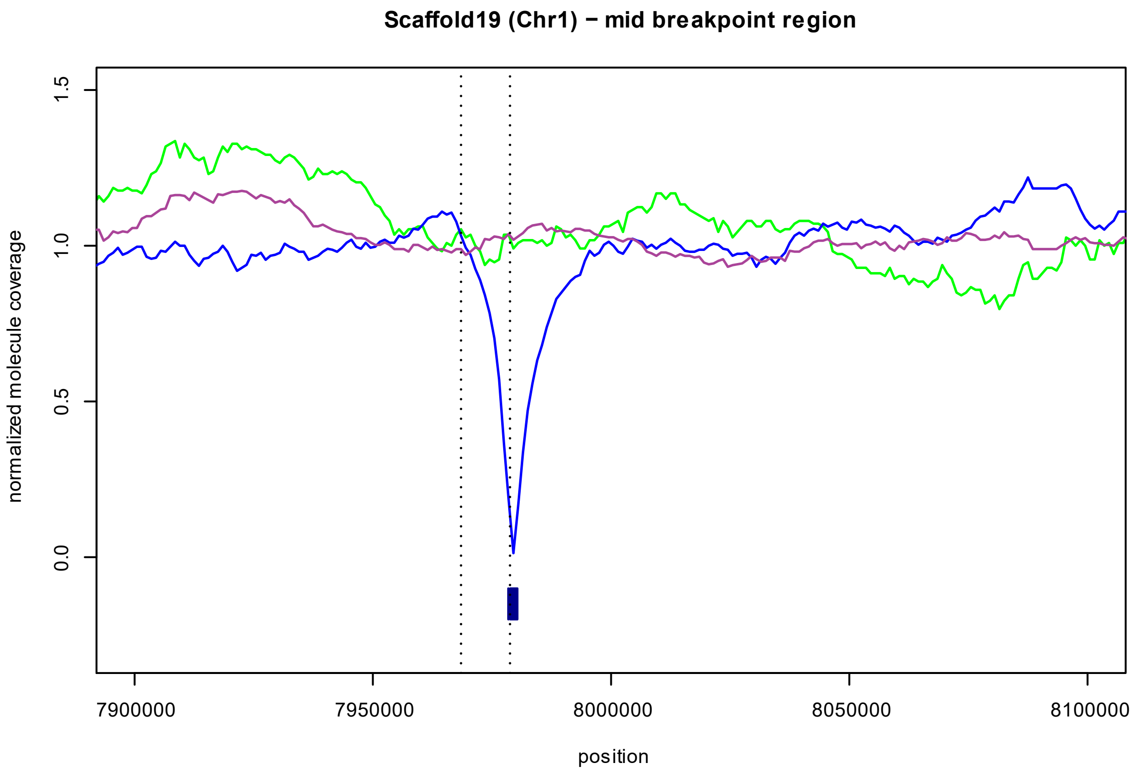
Scaffold19 (Chr1) − mid breakpoint region
normalized molecule coverage
0.0
0.5
1.0
1.5
7900000
7950000
8000000
8050000
8100000
position

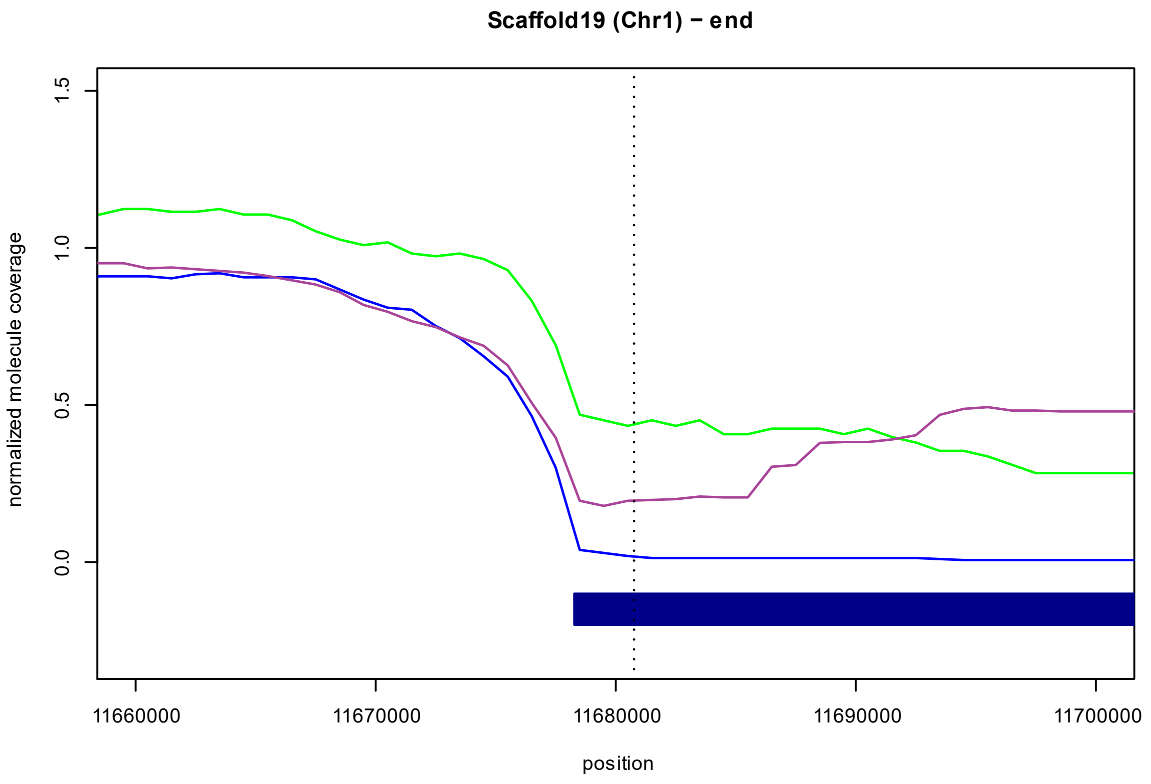
Scaffold19 (Chr1) − end
normalized molecule coverage
0.0
0.5
1.0
1.5
11660000
11670000
11680000
11690000
11700000
position

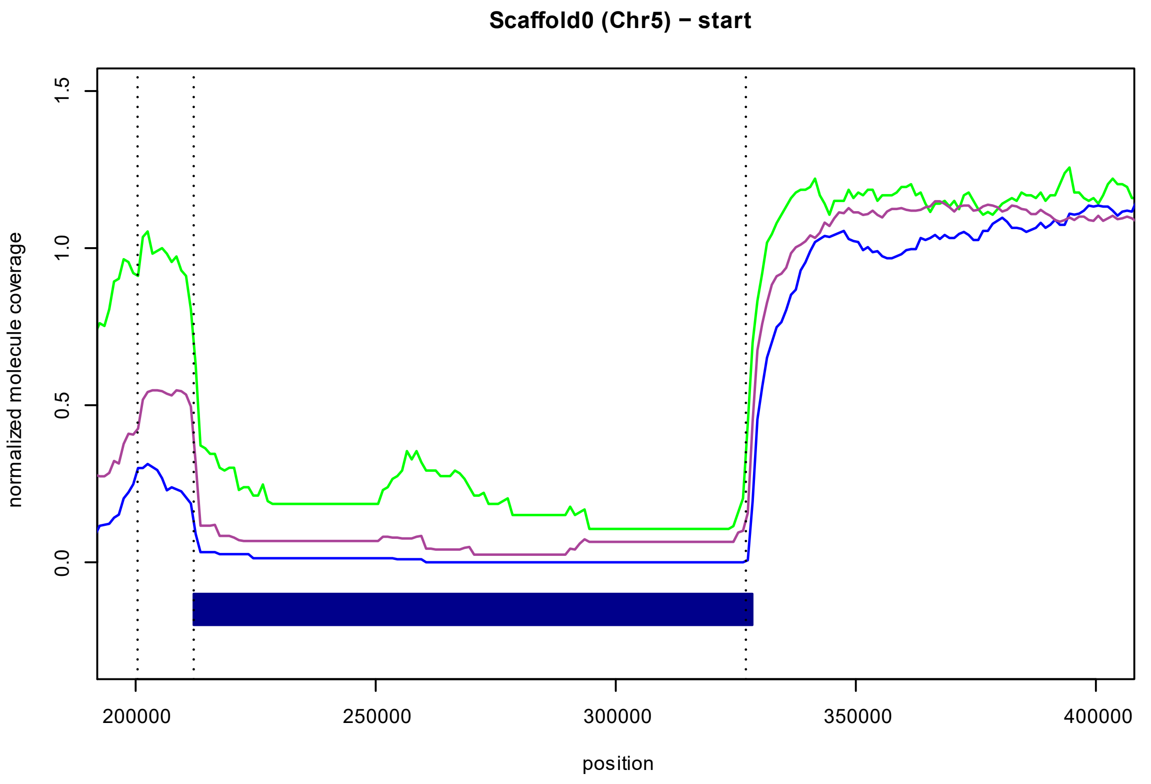
Scaffold0 (Chr5) − start
normalized molecule coverage
0.0
0.5
1.0
1.5
200000
250000
300000
350000
400000
position

Scaffold0 (Chr5) − mid part

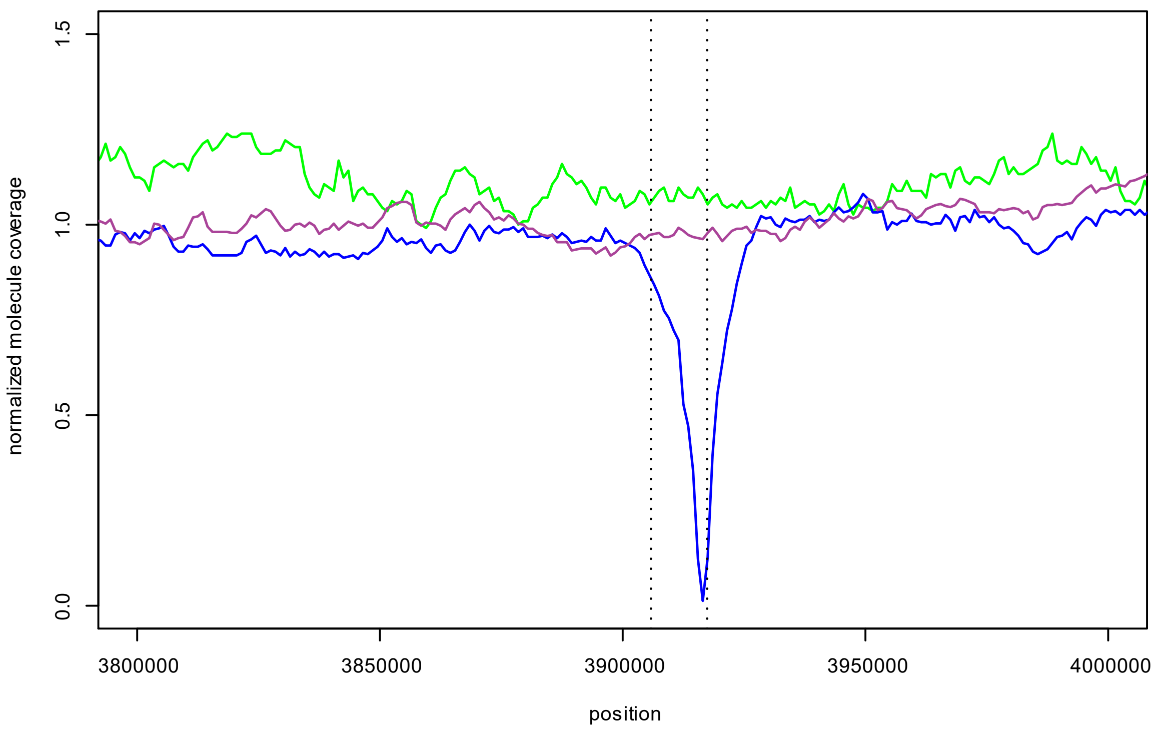

Scaffold0 (Chr5) − end part

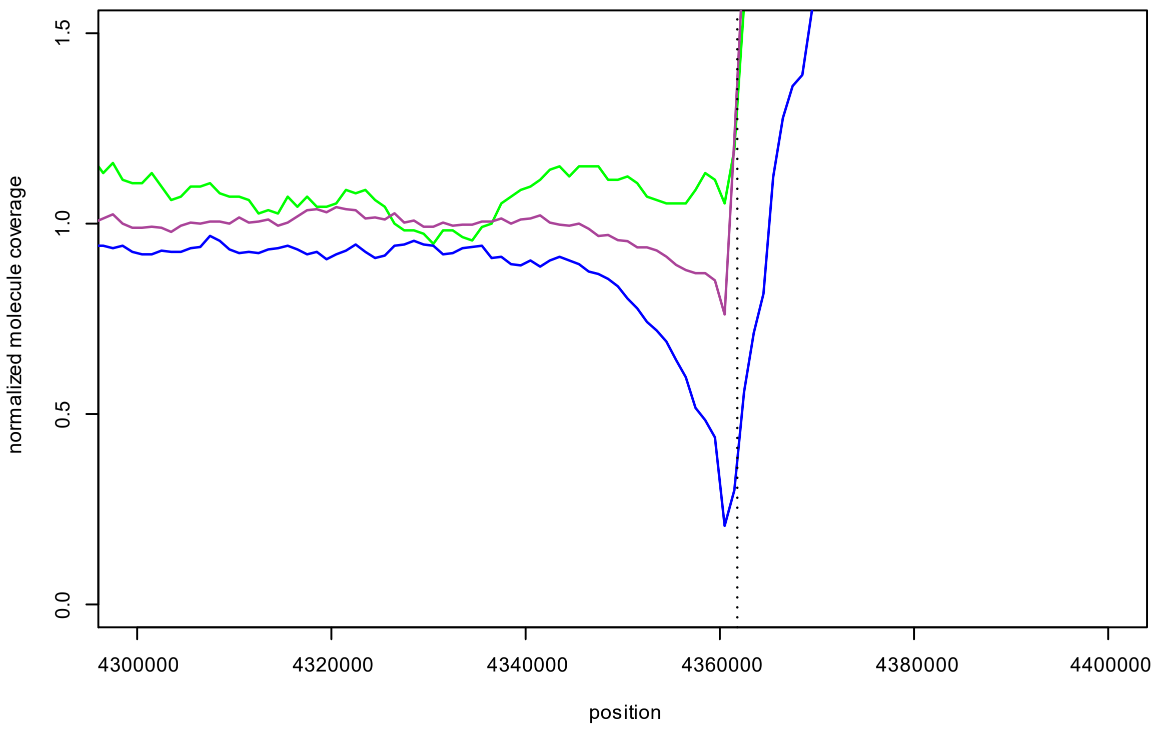

Supplementary Figure 2. Normalized 10x chromium molecule coverage in breakpoint regions in the divergent regions on chromosomes 1 and 5 in the southern willow warbler assembly. Green represents coverage for the southern willow warbler sample, blue the northern willow warbler sample and magenta the additional willow warbler sample that is homozygous southern for the divergent regions on chromosome 1 and 5. Normalized molecule coverage refers to the number of 10x chromium barcodes (molecules) present in non-overlapping 1 kb windows, which has been normalized by the median 1 kb molecule coverage for the sample in each of the divergent regions. Only barcodes associated with molecules that are at least 10 kb have been included. Dotted lines refer to the endpoints of the alignments of scaffolds from the assembly of the other northern sample. Dark blue boxes represent intervals containing tandem repeat arrays.

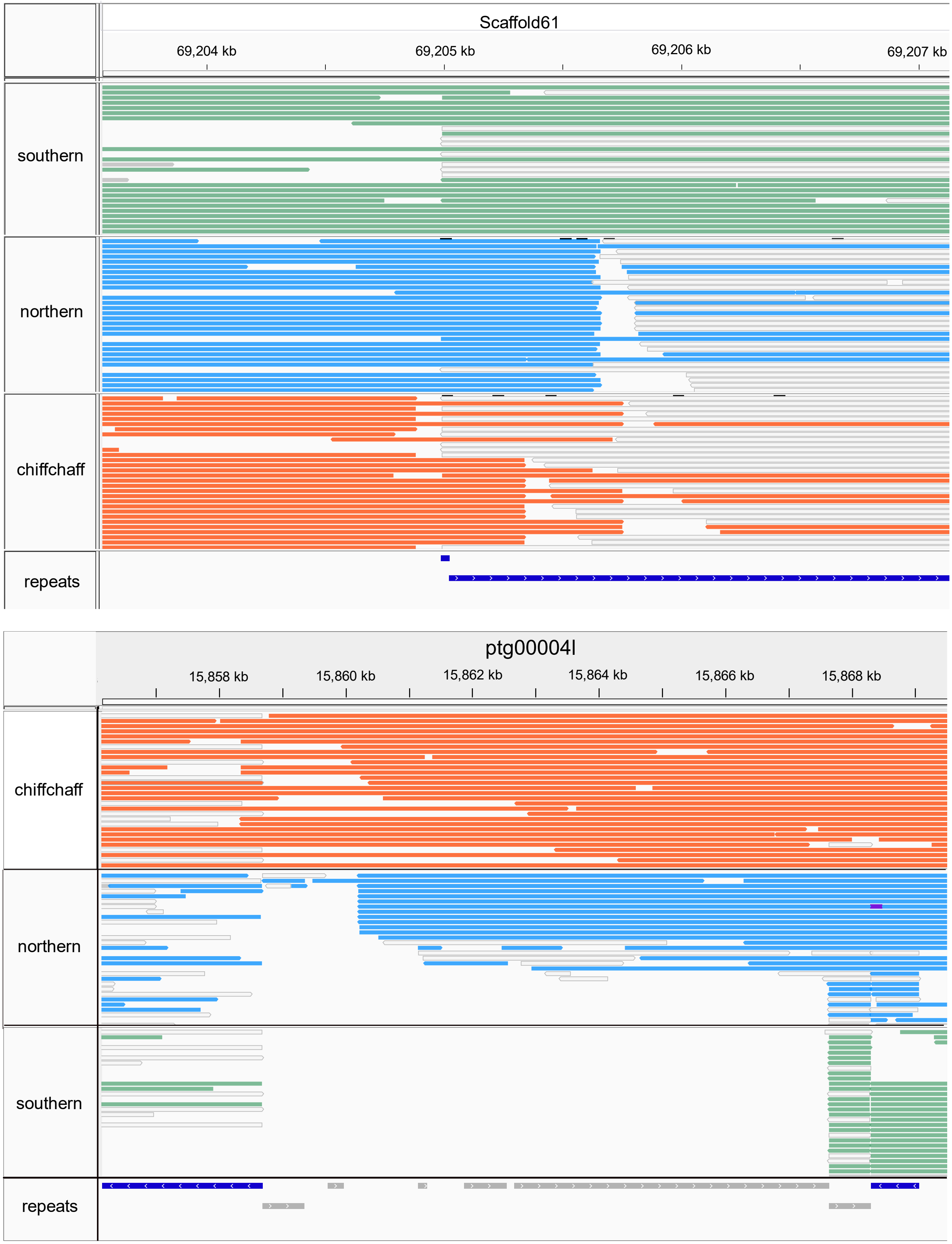
Scaffold61
69,204 kb
69,205 kb
69,206 kb
69,207 kb
southern
northern
chiffchaff
repeats
ptg000004l
15,858 kb
15,860 kb
15,862 kb
15,864 kb
15,866 kb
15,868 kb
chiffchaff
northern
southern
repeats

Supplementary Figure 3. Long read data from the southern willow warbler (green), northern willow warbler (blue) and chiffchaff (orange) mapped to the start of the divergent region on chromosome 3 in the southern willow warbler assembly (Scaffold61) and in the chiffchaff assembly (Contig ptg000040l). Reads with a white filling have a mapping quality of zero and tandem repeat intervals have been highlighted in dark blue. In the southern assembly, northern willow warbler and chiffchaff reads could not be properly aligned beyond the start of the tandem repeat array. In the chiffchaff assembly, the southern willow warbler reads could not be aligned in between the ~9.7 kb interval containing mostly LTR/ERVL repeats between two tandem repeat arrays. In contrast, northern willow warbler reads could be aligned through most of the region, except for the interval just before the first tandem repeat array.

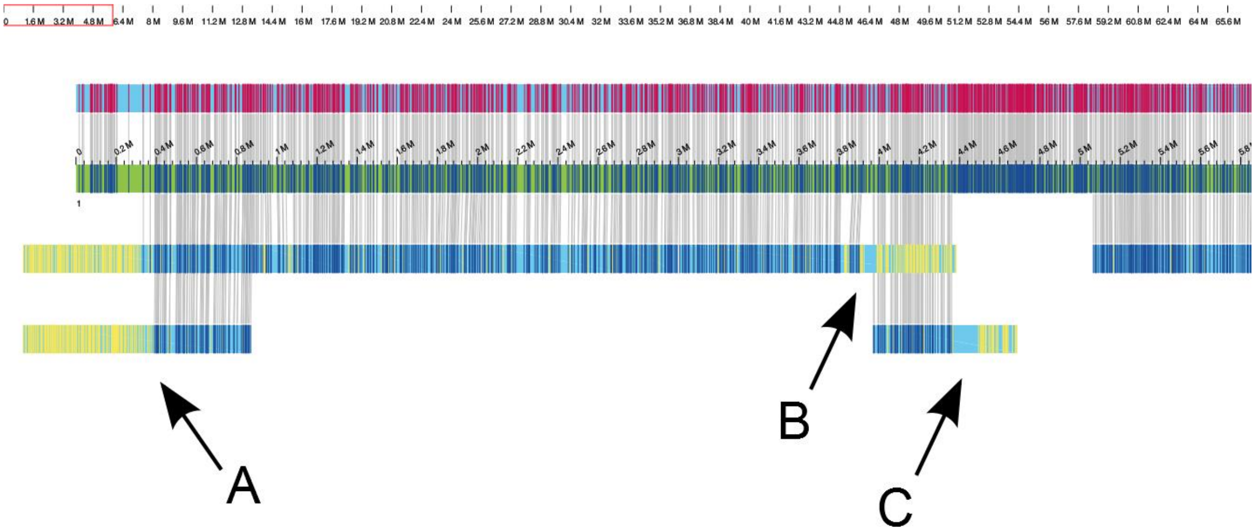

Supplementary Figure 4. Hybrid assembly between the northern optical map and the southern assembly for the divergent region on chromosome 5 (Scaffold0) where no cuts supported by the optical map have been allowed. The upper blue segment represents the optical map of the southern assembly (blue), the green segment the hybrid assembly between the northern assembly and the southern optical map and the blue segments at the bottom, the optical map (contigs) of the northern sample. Matching nicking sites between the northern optical map and the southern genome are connected with vertical lines and highlighted with black bars in each contig. Yellow bars within contigs represent unmatched nicking sites. A) The first breakpoint region with no match before 335 kb. The non-matching part has a nicking site pattern that is reminiscent of that found upstream of the divergent region, around 4.3 Mb. B) Second breakpoint region at around 3910 kb, where the shift occurs between inverted and collinear segments. C) Second collinear interval (3970 kb-4361 kb), with a different optical map contig compared to B), which has been aligned in the reverse orientation but shows the same structural difference to the southern assembly. Both contigs contain a large interval with no nicking sites, which is predicted to correspond to interval around A) in the southern assembly.

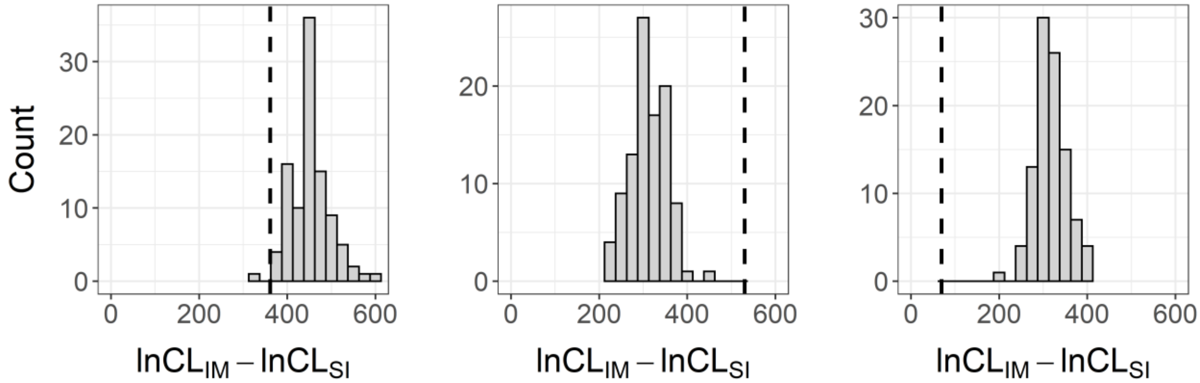

Supplementary Figure 5. Histograms of improvement in composite likelihood (CL) when optimizing 100 simulated strict isolation (SI) datasets under an isolation with migration (IM) model rather than SI. Plots, left to right, are for the chromosome 1, 3, and 5 regions respectively. The dashed lines represent the improvement in the real data, showing that only the chromosome 3 region has an improvement in CL which is greater than expected when there is no migration.

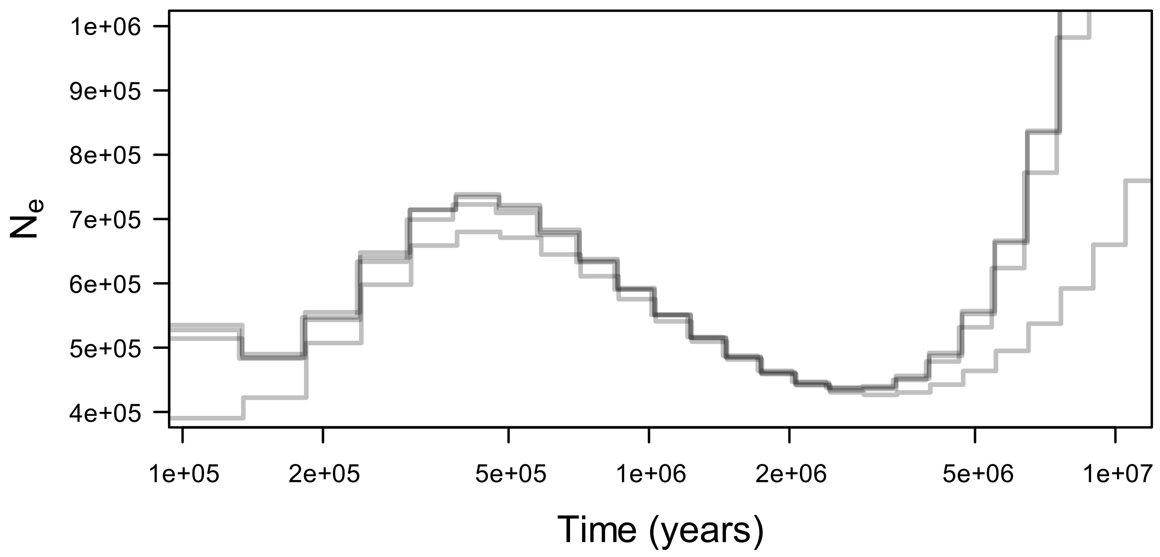

Supplementary Figure 6. Estimates of genome-wide N_e over time inferred from MSMC2 for four high-coverage willow warbler samples. For this analysis, intervals overlapping the divergent regions as well as scaffold shorter than 500 kb have been excluded. Time, between 100KYA and 10MYA, is plotted on a log10 axis.

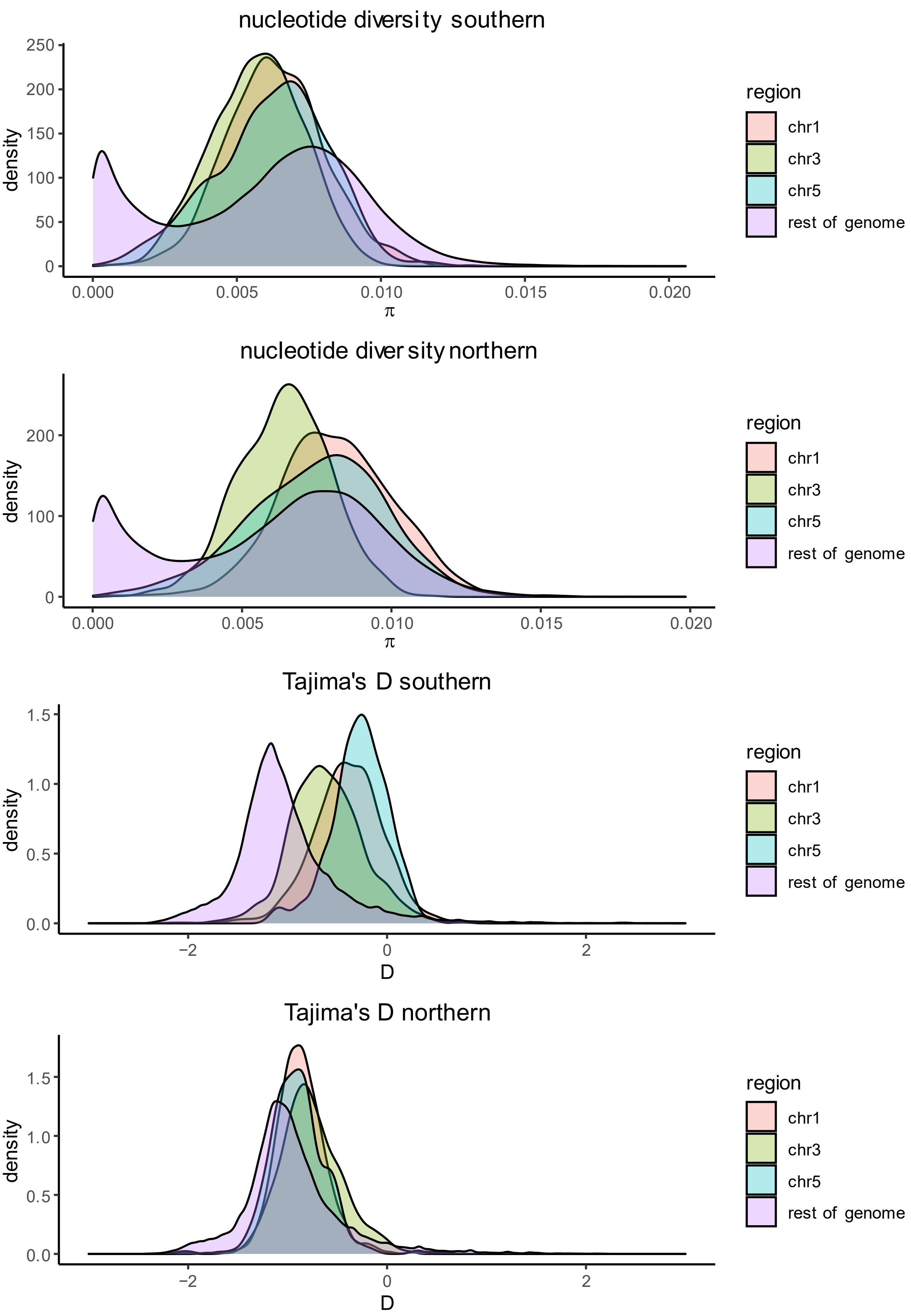
nucleotide diversity southern
density
π
region
chr1
chr3
chr5
rest of genome
nucleotide diversity northern
density
π
region
chr1
chr3
chr5
rest of genome
Tajima's D southern
density
D
region
chr1
chr3
chr5
rest of genome
Tajima's D northern
density
D
region
chr1
chr3
chr5
rest of genome

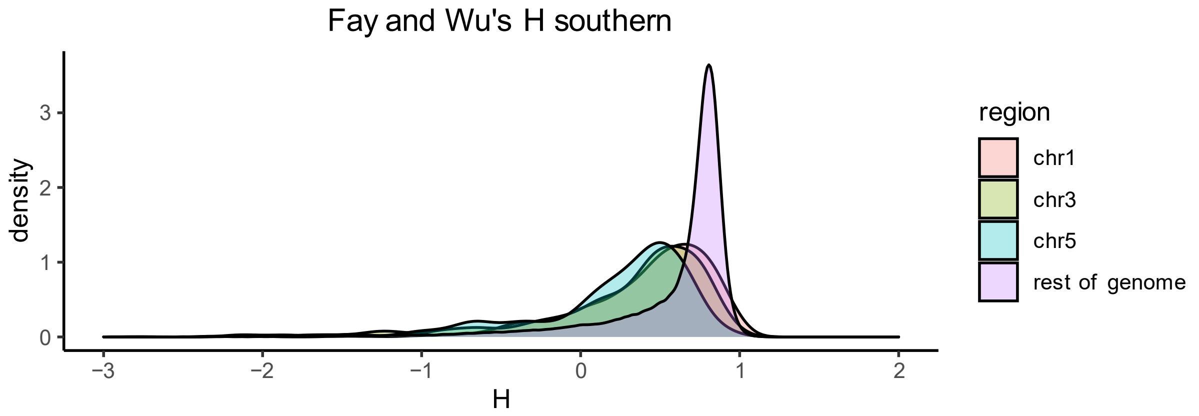
Fay and Wu's H southern
density
H
region
chr1
chr3
chr5
rest of genome

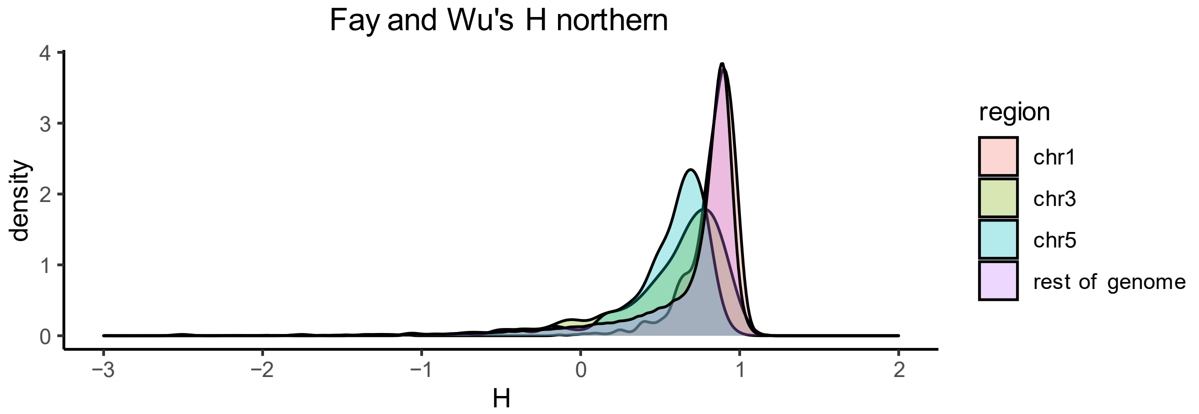
Fay and Wu's H northern
density
H
region
chr1
chr3
chr5
rest of genome

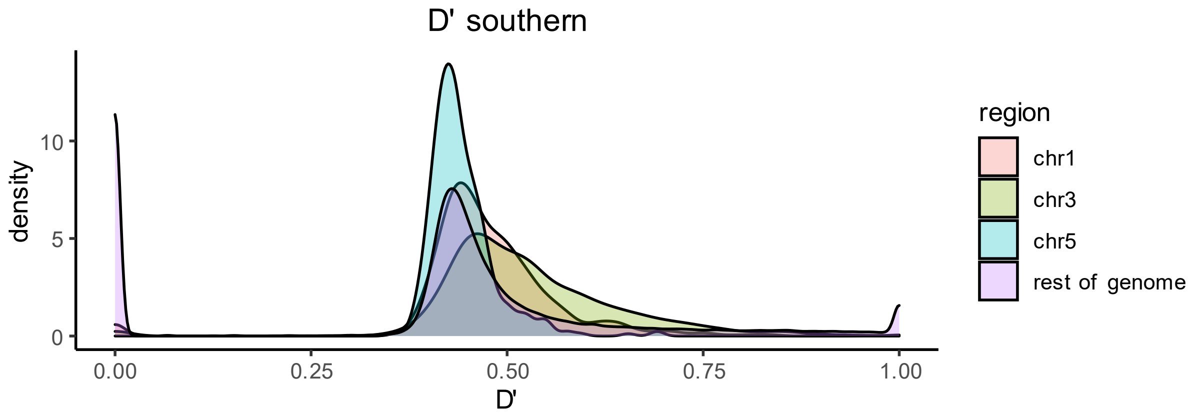
D' southern
density
D'
region
chr1
chr3
chr5
rest of genome

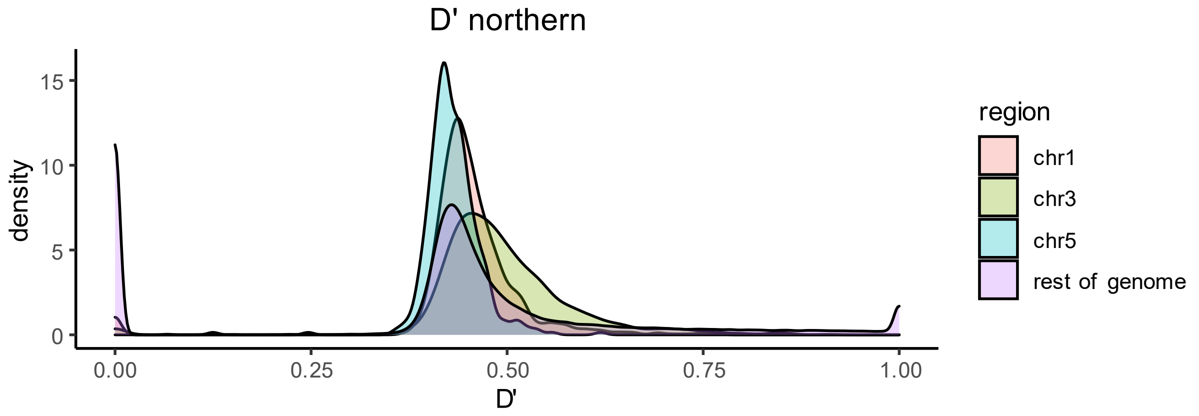
D' northern
density
D'
region
chr1
chr3
chr5
rest of genome

Supplementary Figure 7. Density plots of genetic summary statistics (nucleotide diversity, Tajima's D, Fay and Wu's H and D') in non-overlapping 10 kb windows for pure northern (N=7) and pure southern (N=10) resequenced samples (Supplementary Table 4) in each divergent chromosome region and in the rest of the genome. For D', window-based estimates represent means among pairwise comparisons of SNPs at least 1000 bp from each other and that have a minor allele frequency of at least 0.2 As D' is sensitive to sample size, we randomly removed three southern samples (0G03, 0G04 and UK) to get even sample sizes.

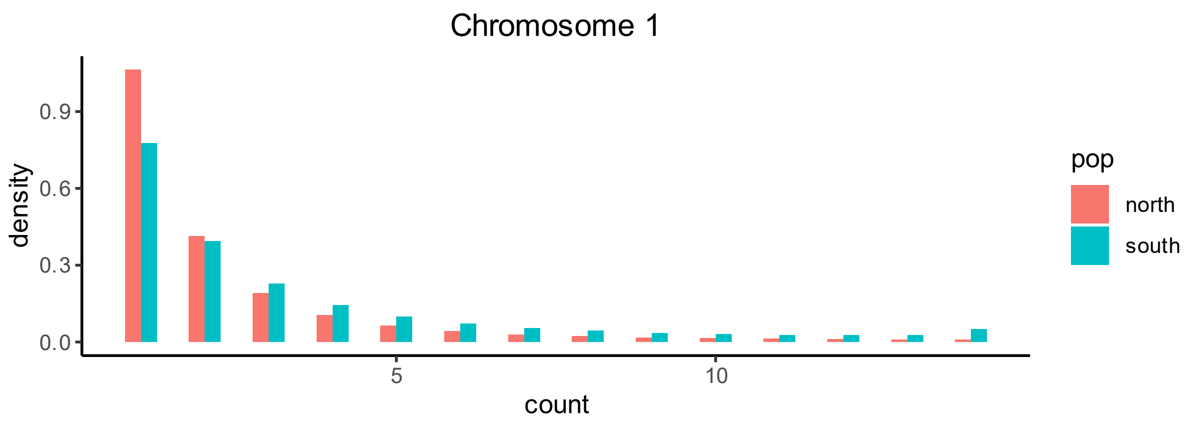
Chromosome 1
density
0.0
0.3
0.6
0.9
5
10
count
pop
north
south

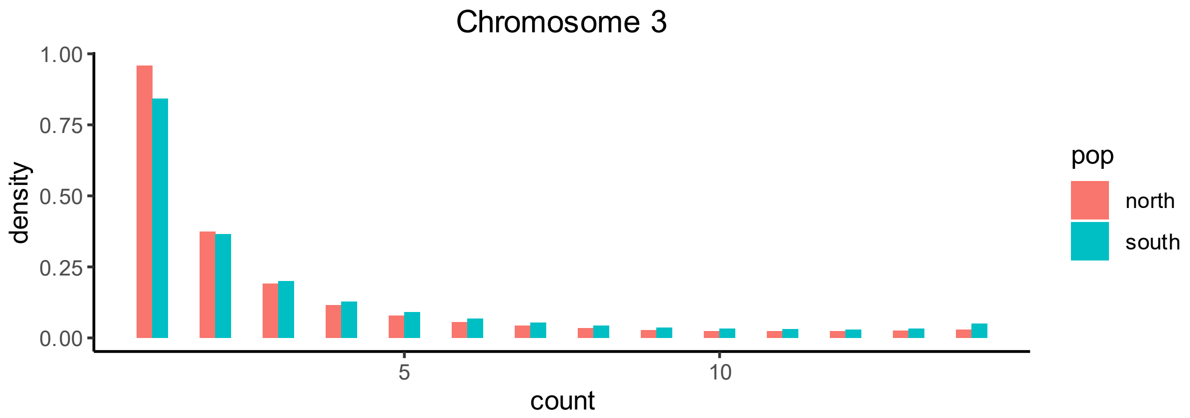
Chromosome 3
density
0.00
0.25
0.50
0.75
1.00
5
10
count
pop
north
south

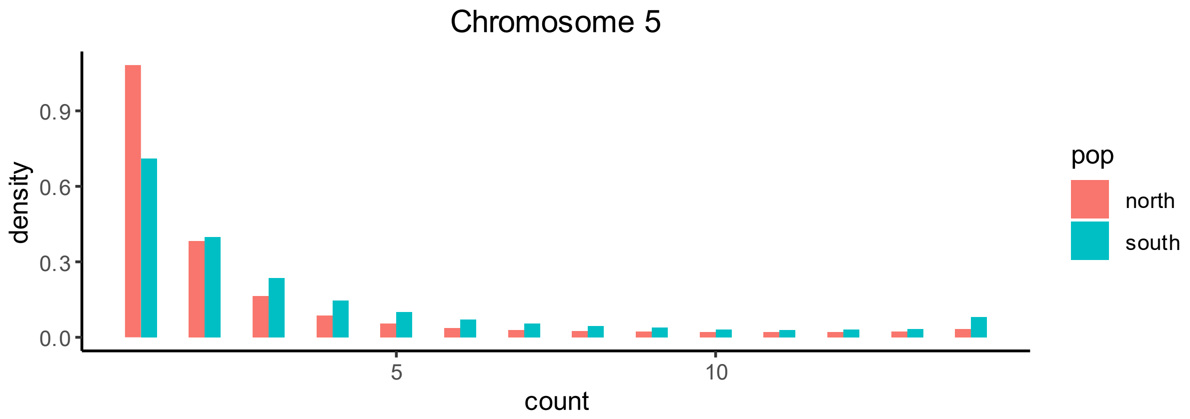
Chromosome 5
density
0.0
0.3
0.6
0.9
5
10
count
pop
north
south

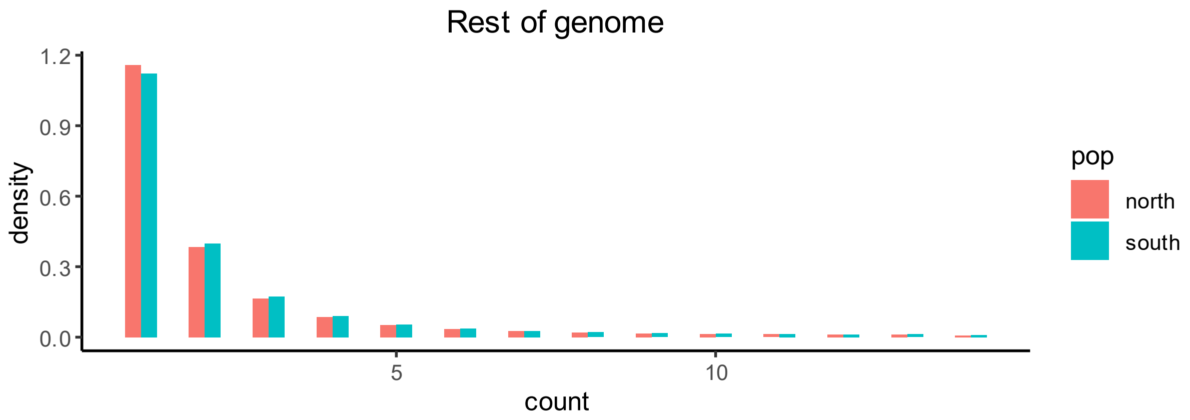
Rest of genome
density
0.0
0.3
0.6
0.9
1.2
5
10
count
pop
north
south

Supplementary Figure 8. Allele frequency spectrum of derived alleles between pure northern and pure southern resequenced samples (Supplementary Table 4) for each of the divergent chromosome regions and the rest of the genome. To simplify the contrast between the southern and northern willow warblers, we randomly removed three southern samples (0G03, 0G04 and UK06) to get even sample sizes (N=7).

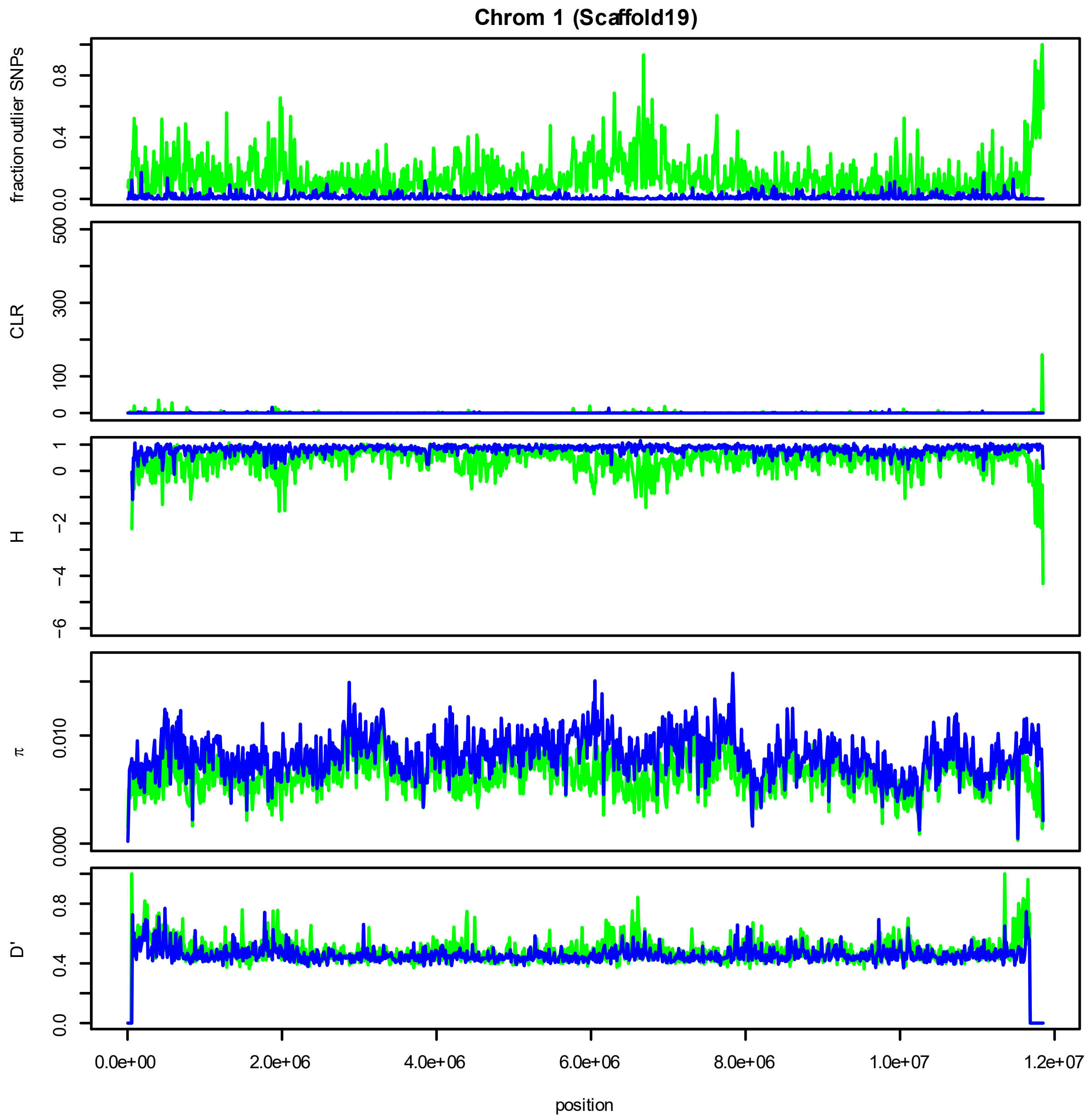
Chrom 1 (Scaffold19)
fraction outlier SNPs
0.0
0.4
0.8
CLR
0
100
300
500
H
1
0
−2
−4
−6
π
0.000
0.010
D'
0.0
0.4
0.8
0.0e+00
2.0e+06
4.0e+06
6.0e+06
8.0e+06
1.0e+07
1.2e+07
position

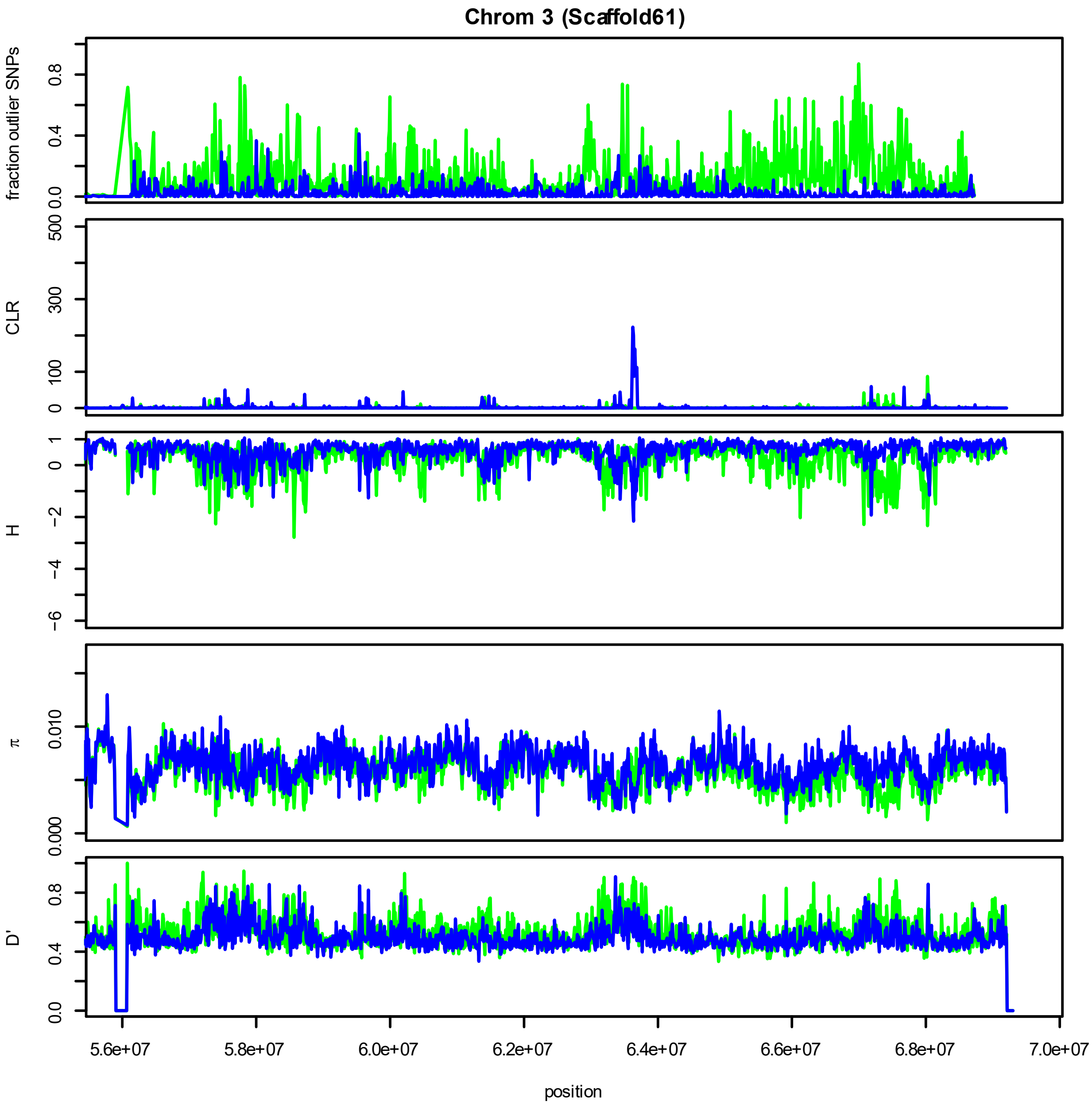

Chrom 3 (Scaffold61)
fraction outlier SNPs
0.0
0.4
0.8
CLR
0
100
300
500
H
1
0
−2
−4
−6
π
0.000
0.010
D'
0.0
0.4
0.8
5.6e+07
5.8e+07
6.0e+07
6.2e+07
6.4e+07
6.6e+07
6.8e+07
7.0e+07
position

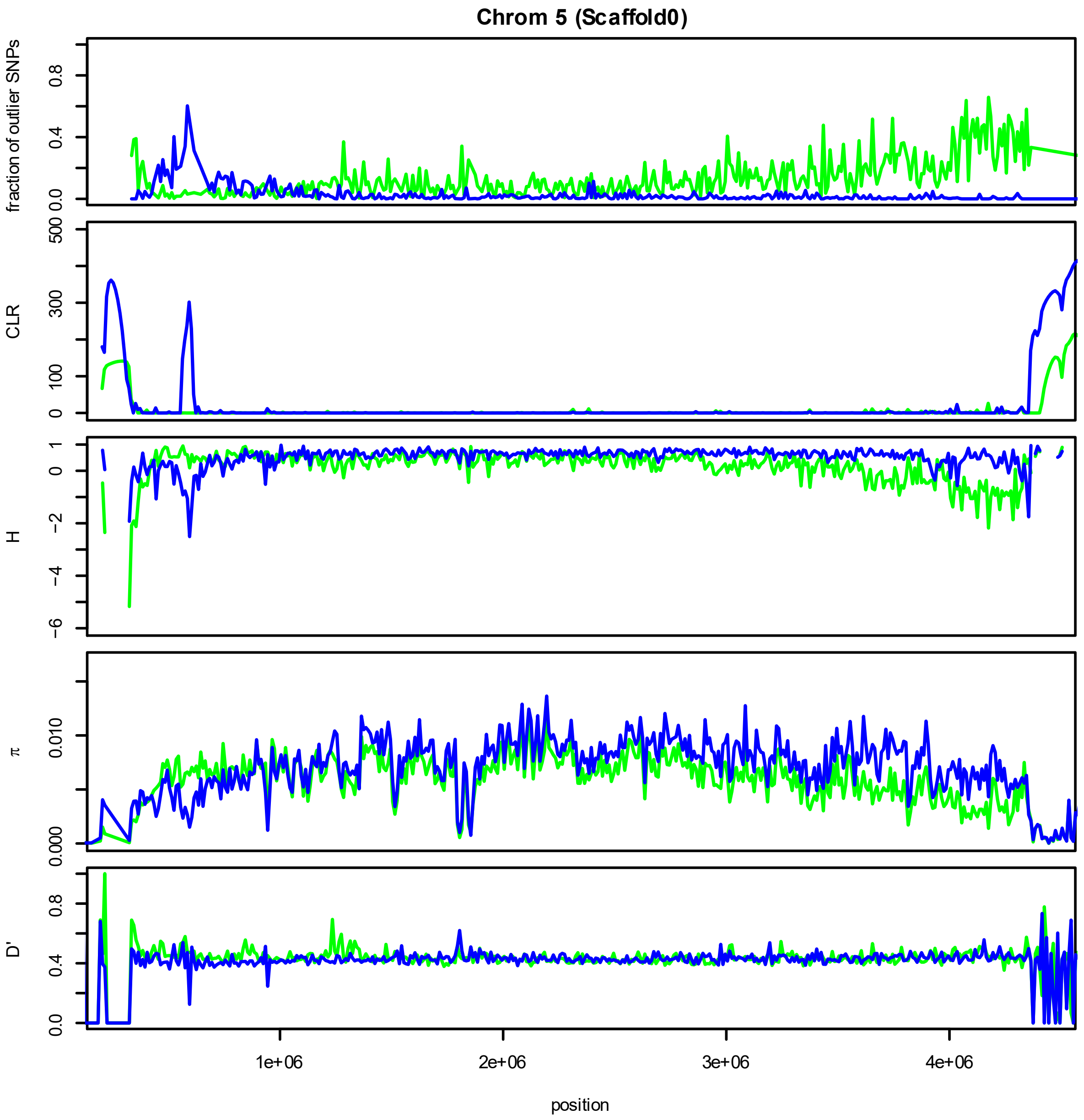

Supplementary Figure 9. Genetic statistics (XP-nsl, Sweepfinder2 CLR, Fay and Wu's H, nucleotide diversity and D') in 10 kb non-overlapping windows for pure northern (blue, N=7) and pure southern resequenced samples (green, N=10) (Supplementary Table 4) in each divergent region. XP-nsl estimates represent fraction of outlier SNPs (|normalized value| > 2) in each window, where green and blue quantify an extended reduction of diversity in southern and northern samples, respectively. Linkage disequilibrium (D') estimates represent means among pairwise comparisons of SNPs at least 1000 bp from each other and that have a minor allele frequency of at least 0.2

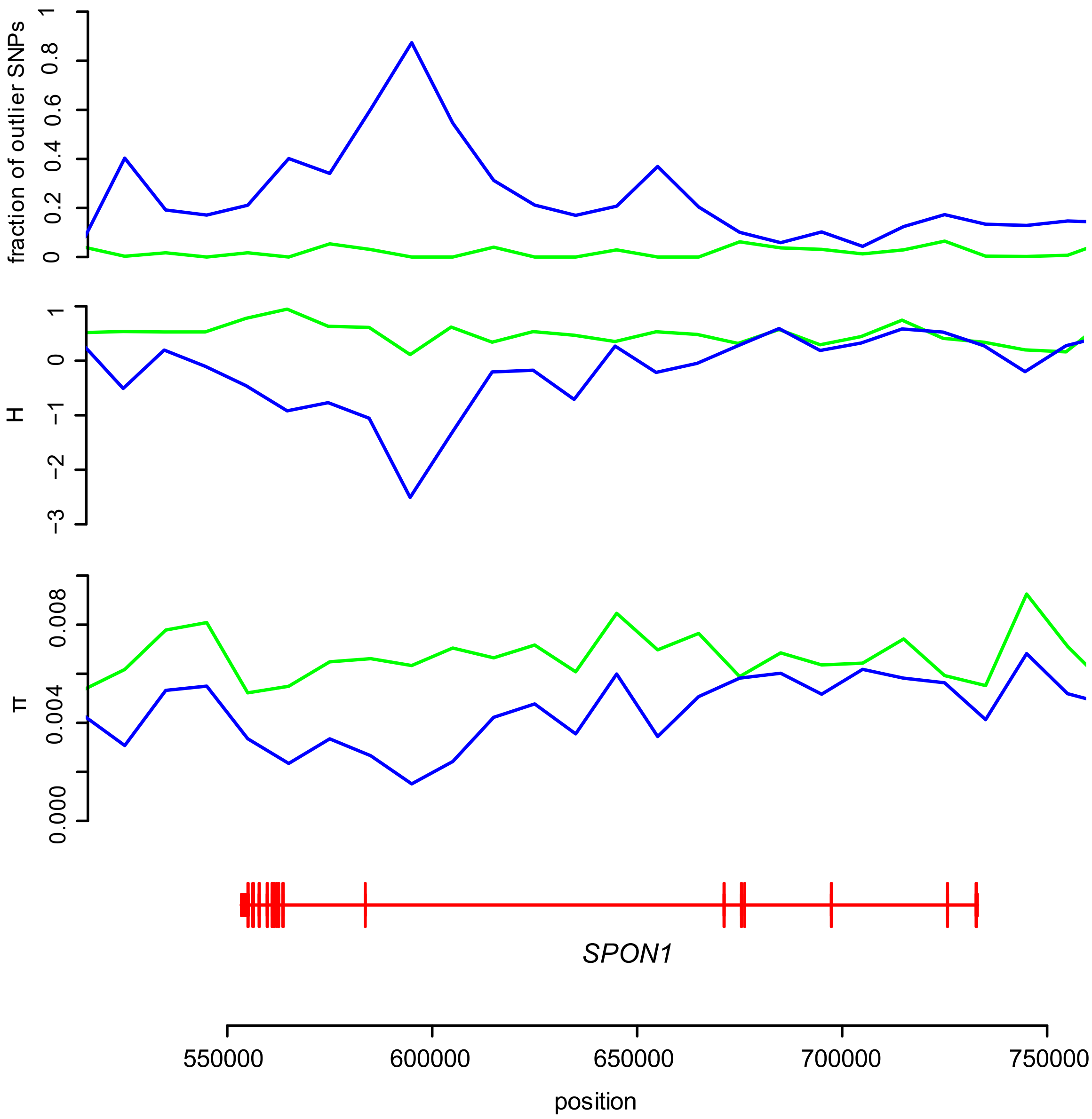

Supplementary Figure 10. Positive selection signatures for northern willow warblers in the *SPON1* gene, which is located at the start of the divergent region on chromosome 5 (Scaffold0). From top to bottom are non-overlapping 10 kb sliding window estimates of XP-nsl, composite likelihood ratio (CLR) statistic from Sweepfinder2 and nucleotide diversity for pure northern (blue, N=7) and pure southern (green, N=10) samples. XP-nsl is here quantified as the proportion of SNPs in each window that are genome-wide outliers (|normalized score| > 2) and either suggesting an extended reduced diversity signal in the southern (green) or northern samples (blue).

Supplementary Table 1. Assembly statistics. Size measures (e.g., N50) are provided in Mb. For scaffolded assemblies contigs are defined as sequences separated by at least 25 Ns. For the Busco analysis, C refers to the number of complete orthologues and S and D, the number of those that are found as single-copy and duplicated genes, respectively. F refers to fragmented genes and M missing genes.

**Assembly**	**Contigs**					**Scaffolds**						**Busco Aves (N=4915)**				
	**N**	**Size**	**N50**	**L50**	**Max**	**N**	**Size**	**N50**	**L50**	**Max**	**%N**	**C**	**S**	**D**	**F**	**M**
Northern de novo assembly																
Primary 30x unzipped contigs	2717	1237	2	154	11.1	N/A	N/A	N/A	N/A	N/A	N/A	4421 (89.9%)	4154 (84.5%)	267 (5.4%)	249 (5.1%)	245 (5.0%)
Primary 40x unzipped contigs	2737	1286	2.1	136	13	N/A	N/A	N/A	N/A	N/A	N/A	4485 (91.3%)	4201 (85.5%)	284 (5.8%)	241 (4.9%)	189 (3.8%)
Primary 40x unzipped contigs + pilon	2737	1286	2.1	136	13	N/A	N/A	N/A	N/A	N/A	N/A	4572 (93.0%)	4206 (85.6%)	366 (7.4%)	190 (3.9%)	153 (3.1%)
Supercontigs (including bionano-split contigs)	2422	1226	6.1	41	35.8	N/A	N/A	N/A	N/A	N/A	N/A	4539 (92.4%)	4315 (87.8%)	224 (4.6%)	200 (4.1%)	176 (3.5%)
Supercontigs with removed haplotigs	1187	1113	7.9	33	35.8	N/A	N/A	N/A	N/A	N/A	N/A	4501 (91.6%)	4435 (90.2%)	66 (1.3%)	222 (4.5%)	192 (3.9%)
Supercontigs with removed haplotigs + arcs	1187	1113	7.9	33	35.8	739	1121	16.4	18	53.2	0.64	4508 (91.7%)	4447 (90.5%)	61 (1.2%)	214 (4.4%)	193 (3.9%)
Supercontigs with removed haplotigs + arcs + bionano	7278	1112	7.9	33	35.8	495	1159	17.1	17	68.9	4.04	4507 (91.7%)	4442 (90.4%)	65 (1.3%)	221 (4.5%)	187 (3.8%)
Supercontigs with removed haplotigs + arcs + bionano + pbjelly	1134	1115	9.3	30	42	495	1160	17.1	17	68.9	3.92	4507 (91.7%)	4442 (90.4%)	65 (1.3%)	221 (4.5%)	187 (3.8%)
Supercontigs with removed haplotigs + arcs + bionano + pbjelly+pilon	1134	1114	9.3	30	42	495	1160	17.1	17	68.9	3.92	4625 (94.1%)	4572 (93.1%)	53 (1.1%)	158 (3.2%)	132 (2.7%)
Final assembly	1128	1114	9.3	30	42	497	1159	16.7	18	67.0	3.87	4625 (94.1%)	4572 (93.1%)	53 (1.1%)	158 (3.2%)	132 (2.7%)
Southern de novo assembly																
Hifiasm	540	1247	34.7	11	95	N/A	N/A	N/A	N/A	N/A	N/A	4674 (95.1%)	4596 (93.5%)	78 (1.6%)	146 (3.0%)	95 (1.9%)

Hifiasm + bionano + filt (final)	986	1247	26.3	14	69.3	547	1249	34.1	12	90	0.21	4680 (95.2%)	4606 (93.7%)	74 (1.5%)	141 (2.9%)	94 (1.9%)
Chiffchaff																
Hifiasm	517	1254	28.1	13	96.3	N/A	N/A	N/A	N/A	N/A	N/A	4669 (95.0%)	4576 (93.1%)	93 (1.9%)	151 (3.1%)	95 (1.9%)
Hifiasm + filt (final)	517	1248	28.1	13	96.3	N/A	N/A	N/A	N/A	N/A	N/A	4669 (95.0%)	4603 (93.7%)	66 (1.3%)	153 (3.1%)	93 (1.9%)

Supplementary Table 2. Repeat content in the northern willow warbler assembly, southern willow warbler assembly, chiffchaff assembly and the previous northern willow warbler short-read assembly (Lundberg et al. 2017). For a more representative comparison we used the repeat library of the southern assembly to identify repeats in the previous short-read genome assembly.

	**Northern assembly**	**Southern assembly**	**Chiffchaff**	**Lundberg *et al.* 2017**
GC	42.65%	43.84%	43.54%	41.96%
Masked bases	172032011 bp (14.84 %)	261870906 bp (20.96 %)	264782513 bp (21.23 %)	103320476 bp (9.69 %)
SINEs	7799 / 934063 bp (0.08 %)	8468 / 955123 (0.08%)	6816 / 848082 bp (0.07 %)	7732 / 925074 bp (0.09%)
ALUs	0 / 0 bp (0.00 %)	0 / 0 bp (0.00 %)	0 / 0 bp (0.00 %)	0 / 0 bp (0.00%)
MIRs	3144 / 344796 bp (0.03 %)	3162 / 347253 bp (0.03 %)	3119 / 344078 bp (0.03 %)	3113 / 339940 bp (0.03 %)
LINEs	151372 / 47345684 bp (4.08 %)	160726 / 53274736 bp (4.26 %)	160193 / 52490333 bp (4.21 %)	139434 / 40653367 bp (3.81 %)
LINE1	78 / 17852 bp (0.00 %)	80 / 18185 (0.00 %)	426 / 320689 bp (0.03 %)	77 / 17352 bp (0.00 %)
LINE2	2196 / 399350 bp (0.03 %)	2200 / 394836 bp (0.03 %)	1655 / 336472 bp (0.03 %)	2173 / 394424 bp (0.04 %)
L3/CR1	143554 / 45431238 bp (3.92 %)	152201 / 50831847 bp (4.07 %)	150393 / 49270575 bp (3.95 %)	133778 / 39916957 bp (3.74 %)
LTR elements	87246 / 55670811 bp (4.80 %)	101321 / 69971355 bp (5.60 %)	111405 / 115207660 bp (9.24 %)	62565 / 29457212 bp (2.76 %)
ERVL	44228 / 24827029 bp (2.14 %)	48682 / 28023260 bp (2.24 %)	54066 / 34906680 bp (2.80 %)	33726 / 16860377 bp (1.58 %)
ERVL-MaLRs	0 / 0 bp (0.00 %)	0 / 0 bp (0.00 %)	0 / 0 bp (0.00 %)	0 / 0 bp (0.00 %)
ERV_classI	19085 / 15006029 bp (1.29 %)	23379 / 18784528 bp (1.50 %)	22780 / 18318440 bp (1.47 %)	11473 / 5156808 bp (0.48 %)
ERV_classII	18867 / 11790452 bp (1.02 %)	22164 / 14855344 bp (1.19 %)	30510 / 59377991 bp (4.76 %)	14656 / 6367209 bp (0.60 %)
DNA elements	34494 / 17705493 bp (1.53 %)	37518 / 19720192 bp (1.58 %)	25172 / 7946391 bp (0.64 %)	20461 / 3205395 bp (0.30 %)
hAT-Charlie	165 / 51112 bp (0.00 %)	161 / 50769 (0.00 %)	166 / 52468 bp (0.00 %)	160 / 50734 bp (0.00 %)
TcMar-Tigger	265 / 46372 bp (0.00 %)	259 / 46024 bp (0.00 %)	271 / 47481 bp (0.00 %)	267 / 46564 bp (0.00 %)
Unclassified	51779 / 22692495 bp (1.96 %)	68749 / 79466852 bp (6.36 %)	58419 / 54893638 bp (4.40 %)	29687 / 10044218 bp (0.94 %)
Total interspersed repeats	144348546 bp (12.45 %)	223388258 bp (17.88 %)	231386104 bp (18.55 %)	84285266 bp (7.90 %)

Supplementary Table 3. RNAseq libraries used for gene annotation. All samples are derived from whole brain tissue. Accession refers to accession number in the Sequence Read Archive.

Sample name	Accession	Sample description	Raw read pairs	Trimmed read pairs
08A02	SRX9729390	Adult male breeding, Silvåkra, Sweden	35 888 086	35 723 400
08A04	SRX9729391	Adult male breeding, Silvåkra, Sweden	41 672 026	41 442 300
08G05	SRX9729392	Adult male breeding, Tångböle, Sweden	43 411 334	43 154 635
08xA10	SRX9729393	Juvenile male migrating, Stensoffa, Sweden	36 588 683	36 426 124
08xB03	SRX9729394	Juvenile male migrating, Skeppshamn, Sweden	36 676 212	36 472 877
08xB13	SRX9729395	Adult male migrating, Skeppshamn, Sweden	42 623 335	42 385 992

Supplementary Table 4. Whole-genome resequencing libraries and 10x chromium linked read libraries. The first 18 resequencing samples were used in Lundberg et al. (2017). Divergent region genotypes are the genotypes for the regions on chromosomes 1, 3 and 5, where S and N denote homozygous southern and northern, respectively, and H heterozygous southern/northern. Samples marked with * were used in the divergence time analyses. Mean coverage has been calculated for sites in the northern genome with nonzero coverage and excluding alignments with mapping quality < 1. Accession refers to accession number in the Sequence Read Archive.

**Sample**	**Accession**	**Sample type**	**Divergent chromosome region genotype**	**Sample location**	**Library type**	**Sequencing setup**	**Mean cov**
Whole-genome resequencing							
0G03	SRX1764074-SRX1764079	*trochilus* (male)	SSS	Stensoffa, Sweden, 55.7°N, 13.4°E	670 bp, Truseq DNA PCR free	Hiseq2000, 2x100 bp	7
0G04	SRX1764080-SRX1764085	*trochilus* (male)	SSS	Stensoffa, Sweden, 55.7°N, 13.4°E	670 bp, Truseq DNA PCR free	Hiseq2000, 2x100 bp	6
0G10	SRX1764086-SRX1764091	*trochilus* (male)	SSS	Stensoffa, Sweden, 55.7°N, 13.4°E	670 bp, Truseq DNA PCR free	Hiseq2000, 2x100 bp	7
0J01	SRX1764092-SRX1764097	*trochilus* (male)	SSS	Smedstorp, Sweden, 59.6°N, 15.0°E	670 bp, Truseq DNA PCR free	Hiseq2000, 2x100 bp	7
1K05	SRX1764098-SRX1764103	*acredula* (male)	NHN	Tornehamn, Sweden, 68.4°N, 18.6°E	670 bp, Truseq DNA PCR free	Hiseq2000, 2x100 bp	8
1K10	SRX1764104-SRX1764109	*acredula* (male)	NNN	Tornehamn, Sweden, 68.4°N, 18.6°E	670 bp, Truseq DNA PCR free	Hiseq2000, 2x100 bp	8
1L17	SRX1764110-SRX1764115	*acredula* (male)	NNN	Stordalen, Sweden, 68.3°N, 19.1°E	670 bp, Truseq DNA PCR free	Hiseq2000, 2x100 bp	8

1L19*	SRX1736538	*acredula* (male)	NNN	Stordalen, Sweden, 68.3°N, 19.1°E	670 bp, Truseq DNA PCR free	Hiseq2500, 2x125 bp	44
1L20	SRX1764116-SRX1764121	*acredula* (male)	NNN	Stordalen, Sweden, 68.3°N, 19.1°E	670 bp, TruSeq DNA PCR free	Hiseq2000, 2x100 bp	7
1M08	SRX1764122-SRX1764127	*acredula* (male)	NNN	Kaisepakte, Sweden, 68.3°N, 19.3°E	670 bp, Truseq DNA PCR free	Hiseq2000, 2x100 bp	7
1O01	SRX1764128-SRX1764133	*acredula* (male)	NNN	Kallax, Sweden, 65.5°N, 22.1°E	670 bp, Truseq DNA PCR free	Hiseq2000, 2x100 bp	14
1O04	SRX1764134-SRX1764139	*acredula* (male)	NSN	Kallax, Sweden, 65.5°N, 22.1°E	670 bp, Truseq DNA PCR free	Hiseq2000, 2x100 bp	14
1O06	SRX1764140-SRX1764145	*acredula* (male)	HSN	Kallax, Sweden, 65.5°N, 22.1°E	670 bp, Truseq DNA PCR free	Hiseq2000, 2x100 bp	11
1P02	SRX1764146-SRX1764151	*trochilus* (male)	SSS	Barkö, Sweden, 60.2°N, 18.2°E	670 bp, Truseq DNA PCR free	Hiseq2000, 2x100 bp	9
3K06	SRX1764152-SRX1764157	*trochilus* (male)	SSH	Gräsmarö, Sweden, 58.3°N,17.0°E	670 bp, Truseq DNA PCR free	Hiseq2000, 2x100 bp	8
7A12	SRX1764170-SRX1764175	*trochilus* (male)	SSS	Haganäs, Sweden, 56.3°N, 14.7°E	670 bp, Truseq DNA PCR free	Hiseq2000, 2x100 bp	7
96A01	SRX1764158-SRX1764163	*trochilus* (male)	SSS	Stensoffa, Sweden, 55.7°N, 13.4°E	670 bp, Truseq DNA PCR free	Hiseq2000, 2x100 bp	7
96B07	SRX1764164-SRX1764169	*trochilus* (male)	SSS	Kvismaren, Sweden, 59.2°N, 15.4°E	670 bp, Truseq DNA PCR free	Hiseq2000, 2x100 bp	8
1A05*	SRX9717361	*trochilus* (female)	SSS	Stensoffa, Sweden, 55.7°N, 13.4°E	350 bp, TruSeq Nano DNA	HiseqX, 2x150 bp	24
1M13*	SRX9738264	*acredula* (female)	NNN	Kaisepakte, Sweden, 68.3°N, 19.3°E	350 bp, TruSeq Nano DNA	HiseqX, 2x150 bp	26
1N12	SRX9719306	*acredula* (female)	NSN	Altajärvi, Sweden, 67.8°N,20.5°E	350 bp, TruSeq Nano DNA	HiseqX, 2x150 bp	20

UK06*	SRX9710091	*trochilus* (female)	SSS	Fife, United Kingdom, 56.2°N, 3.1°W	670 bp, Truseq DNA PCR free	HiseqX, 2x150 bp	29
DW83	SRX9759343	Dusky warbler *Phylloscopus fuscatus* (female)	N/A	Malkachan river, Russia, 59.5°N, 154.1°E	550 bp, Neoprep	HiseqX, 2x150 bp	32
Linked reads							
CP59520 (northern assembly)	SRX9809766	*acredula* (male)	NNN	Krankesjön, Sweden, 55.7°N, 13.4°E	10x chromium	HiseqX 2x150 bp	84
CP59525 (southern assembly)	SRX9804925	*trochilus* (male)	SSS	Krankesjön, Sweden, 55.7°N, 13.4°E	10x chromium	HiseqX 2x150 bp	59
DD81063	SRX14774353	male	SHS	Krankesjön, Sweden, 55.7°N, 13.4°E	10x chromium	NovaSeq6000 2x150 bp	65

Supplementary Table 5. Number of highly differentiated variants between 11 resequenced birds from each subspecies that are located in each divergent region and in the rest of the genome.

F_{ST} threshold	Chr1	Chr3	Chr5	Other (N scaffolds)
0.5	21887 (49.3%)	11027 (24.8%)	9880 (22.3%)	1610 (3.6%, 81 scaffolds)
0.6	12754 (54.2%)	3996 (17.0%)	6432 (27.3%)	342 (1.5%, 53 scaffolds)
0.7	7224 (60.9%)	393 (3.3%)	4098 (34.6%)	140 (1.2%, 24 scaffolds)
0.8	3269 (56.7%)	21 (0.4%)	2416 (41.9%)	58 (1.0%, 10 scaffolds)
0.9	654 (37.0%)	2 (0.1%)	1094 (61.9%)	17 (1.0%, 5 scaffolds)
1	21 (10.8%)	0 (0%)	165 (84.6%)	9 (4.6%, 2 scaffolds)

Supplementary Table 6. Divergence statistics

Region	dxy	pi north	pi south	da	divergence time (years)	RND dusky warbler
Chromosome 1	0.012	0.010	0.008	0.003	511 848	0.409
Chromosome 3	0.010	0.007	0.006	0.003	544 185	0.352
Chromosome 5	0.013	0.011	0.009	0.003	538 641	0.389

Supplementary Table 7. Demographic parameter estimates. Ne: Effective population size, me: migration rate, SI: Strict Isolation, IM2: Isolation with migration model 2.

**chromosome_1 - SI**					
	**Ne_northern**	**Ne_southern**	**Ne_ancestral**	**split_years**	**m_e**
MCL	1 566 413	592 968	273 471	1 197 770	NA
SIM_SD	41 138	10 662	2 672	8 056	NA
SIM_mean	1 658 205	614 056	236 147	1 275 983	NA
Bias	91 792	21 088	-37 325	78 212	NA
**chromosome_3 - IM2**					
	**Ne_northern**	**Ne_southern**	**Ne_ancestral**	**split_years**	**m_e**
MCL	665 708	323 997	176 903	1 302 349	6.53E-07
SIM_SD	19 911	7233	7225	30 667	4.06E-08
SIM_mean	661 595	296 279	89 084	1 617 953	8.11E-07
Bias	-4 113	-27 718	-87 819	315 604	1.58E-07
**chromosome_5 - SI**					
	**Ne_northern**	**Ne_southern**	**Ne_ancestral**	**split_years**	**m_e**
MCL	1 519 746	703 791	318 141	1 218 887	NA
SIM_SD	67 767	21 671	4 123	12 098	NA
SIM_mean	1 639 923	742 029	265 375	1 340 160	NA
Bias	120 177	38 238	-52 766	121 272	NA
**Rest of the genome - SI synced in all populations**					
	Ne	split_years			
MCL	459 177	101 930			

Supplementary Table 8. Gene annotations closest to breakpoints. Breakpoints have been defined as endpoints of alignments of the northern assembly.

**Breakpoint**	**Position**	**Gene model**	**Gene**	**Distance from breakpoint/orientation relative gene start**
Chr1_start	Scaffold19:58075	Phtr_g5241_UFM1	Ubiquitin-fold modifier 1 (*UFM1*)	44.0 kb downstream
Chr1_mid	Scaffold19:7980361	Phtr_g27883_COG3	Component Of Oligomeric Golgi Complex 3 (*COG3*)	6.1 kb upstream
Chr1_end	Scaffold19:11680761	Phtr_g9056_FREM2	FRAS1 Related Extracellular Matrix 2 (*FREM2*)	71.2 kb upstream
Chr3_start	Scaffold61:56069986	Phtr_g20118_COA6	Cytochrome C Oxidase Assembly Factor 6 (*COA6*)	11.6 kb upstream
Chr3_end	Scaffold61:69205658	Phtr_g10669_WTAP	WT1 Associated Protein (*WTAP*)	2.9 kb upstream
Chr5_start	Scaffold0:327097	Phtr_g7229_OLFML1	Olfactomedin Like 1 (*OLFML1*)	3.5 kb downstream
Chr5_mid	Scaffold0:3917392	Phtr_g5159_ANO3	Anoctamin 3 (*ANO3*)	13.4 kb upstream
Chr5_end	Scaffold0:4361809	Phtr_g10614_MAS1-like-2	MAS1 Proto-Oncogene, G Protein-Coupled Receptor-like-2 (*MAS1-like-2*)	1.5 kb upstream

Supplementary Table 9. Highly differentiated variants (F_{ST}≥0.7) between southern and northern homozygotes in each of the divergent chromosome regions that have a predicted impact on the protein-coding sequence of genes. For type, M refers to missense variant, C conservative inframe insertion, S splice region variant and F frameshift variant.

**Scaffold**	**Position**	**FST**	**REF**	**ALT**	**Gene model**	**Gene**	**Type**
Scaffold19	1087641	0.70867	T	G	Phtr_g1064_CCDC169	coiled-coil domain containing 169 (*CCDC169*)	M
Scaffold19	1319577	0.80765	T	C	Phtr_g19298_DCLK1	doublecortin like kinase 1 (*DCLK1*)	M
Scaffold19	2598010	0.71456	C	T	Phtr_g10529_KL	klotho (*KL*)	M
Scaffold19	2885581	0.9501	A	T	Phtr_g5904_N4BP2L2	NEDD4 binding protein 2 like 2 (*N4BP2L2*)	M
Scaffold19	2886086	0.70437	G	A	Phtr_g5904_N4BP2L2	NEDD4 binding protein 2 like 2 (*N4BP2L2*)	M
Scaffold19	2886300	0.83204	A	C	Phtr_g5904_N4BP2L2	NEDD4 binding protein 2 like 2 (*N4BP2L2*)	M
Scaffold19	2886494	0.79167	G	A	Phtr_g5904_N4BP2L2	NEDD4 binding protein 2 like 2 (*N4BP2L2*)	M
Scaffold19	2931630	0.76995	G	A	Phtr_g21712_BRCA2	BRCA2, DNA repair associated (*BRCA2*)	M
Scaffold19	4319909	0.88933	A	G	Phtr_g5007_FLT1	fms related receptor tyrosine kinase 1 (*FLT1*)	M
Scaffold19	4381077	1	C	A	Phtr_g11922_PAN3	PAN3 poly(A) specific ribonuclease subunit (*PAN3*)	M,S
Scaffold19	4577265	0.75667	G	A	Phtr_g11669_POLR1D	DNA-directed RNA polymerases I and III subunit RPAC2 (*POLR1D*)	M
Scaffold19	4692444	0.80097	AT	A	Phtr_g5915_GTF3A	general transcription factor IIIA (*GTF3A*)	F
Scaffold19	4693137	0.77239	T	C	Phtr_g5915_GTF3A	general transcription factor IIIA (*GTF3A*)	M
Scaffold19	4693216	0.7557	G	T	Phtr_g5915_GTF3A	general transcription factor IIIA (*GTF3A*)	M
Scaffold19	4694975	1	T	G	Phtr_g5915_GTF3A	general transcription factor IIIA (*GTF3A*)	M
Scaffold19	5160229	0.71862	T	C	Phtr_g14759_RNF6	ring finger protein 6 (*RNF6*)	M
Scaffold19	5815740	0.81859	G	A	Phtr_g4856_SPATA13	spermatogenesis associated 13 (*SPATA13*)	M
Scaffold19	7312964	0.84314	A	G	Phtr_g6595_CRYL1	crystallin lambda 1 (*CRYL1*)	M
Scaffold19	7514106	0.7114	C	T	Phtr_g26383_ZMYM2	Zinc finger MYM-type protein 2 (*ZMYM2*)	M
Scaffold19	8066066	1	TG	CA	Phtr_g7324_CBY2	Protein chibby homolog 2 (*CBY2*)	M
Scaffold19	8226288	0.79913	T	C	Phtr_g4607_CPB2	carboxypeptidase B2 (*CPB2*)	M

Scaffold19	8999053	0.86762	A	ACCT	Phtr_g21368_RB1	RB transcriptional corepressor 1 (*RB1*)	C
Scaffold19	1.1E+07	0.80936	T	C	Phtr_g19921_CKAP2	cytoskeleton associated protein 2 (*CKAP2*)	M
Scaffold61	5.8E+07	0.76504	C	A	Phtr_g19478_FMN2	formin 2 (*FMN2*)	M
Scaffold61	5.9E+07	0.82696	A	G	Phtr_g17712_WDR64	WD repeat domain 64 (*WDR64*)	M
Scaffold61	5.9E+07	0.78868	T	A	Phtr_g17712_WDR64	WD repeat domain 64 (*WDR64*)	M
Scaffold61	5.9E+07	0.76508	C	G	Phtr_g745_TAB2	TGF-beta activated kinase 1/MAP3K7 binding protein 2 (*TAB2*)	M
Scaffold61	6.1E+07	0.80527	G	A	Phtr_g12364_UTRN	utrophin (*UTRN*)	M
Scaffold61	6.2E+07	0.94191	T	C	Phtr_g22448_MTFR2	mitochondrial fission regulator 2 (*MTFR2*)	M
Scaffold61	6.2E+07	0.7972	C	G	Phtr_g22448_MTFR2	mitochondrial fission regulator 2 (*MTFR2*)	M
Scaffold61	6.2E+07	0.75269	A	T	Phtr_g22448_MTFR2	mitochondrial fission regulator 2 (*MTFR2*)	M
Scaffold61	6.2E+07	0.76818	A	G	Phtr_g17255_KPNA2	Importin subunit alpha-2 (*KPNA2*)	M
Scaffold61	6.2E+07	0.7368	T	C	Phtr_g26752_IFNGR1	interferon gamma receptor 1 (*IFNGR1*)	M
Scaffold61	6.7E+07	0.88617	G	C	Phtr_g28041_SCAF8	SR-related CTD associated factor 8 (*SCAF8*)	M
Scaffold61	6.7E+07	0.75267	T	C	Phtr_g28041_SCAF8	SR-related CTD associated factor 8 (*SCAF8*)	M
Scaffold61	6.7E+07	0.75267	A	G	Phtr_g28041_SCAF8	SR-related CTD associated factor 8 (*SCAF8*)	M
Scaffold61	6.7E+07	0.94344	A	G	Phtr_g28041_SCAF8	SR-related CTD associated factor 8 (*SCAF8*)	M
Scaffold61	6.7E+07	0.7395	T	G	Phtr_g11696_IPCEF1	tohesin exchange factors 1 (*IPCEF1*)	M
Scaffold61	6.8E+07	0.84791	T	G	Phtr_g6597_SYNE1	spectrin repeat containing, nuclear envelope 1 (*SYNE1*)	M
Scaffold61	6.8E+07	0.7166	A	G	Phtr_g6597_SYNE1	spectrin repeat containing, nuclear envelope 1 (*SYNE1*)	M,S
Scaffold61	6.8E+07	0.82696	G	A	Phtr_g6597_SYNE1	spectrin repeat containing, nuclear envelope 1 (*SYNE1*)	M
Scaffold61	6.8E+07	0.71401	A	C	Phtr_g6597_SYNE1	spectrin repeat containing, nuclear envelope 1 (*SYNE1*)	M
Scaffold61	6.8E+07	0.82498	C	G,T	Phtr_g6597_SYNE1	spectrin repeat containing, nuclear envelope 1 (*SYNE1*)	M
Scaffold61	6.8E+07	0.81959	C	A	Phtr_g6597_SYNE1	spectrin repeat containing, nuclear envelope 1 (*SYNE1*)	M

Scaffold61	6.8E+07	0.73021	C	T	Phtr_g6597_SYNE1	spectrin repeat containing, nuclear envelope 1 (*SYNE1*)	M
Scaffold61	6.8E+07	0.80172	C	G	Phtr_g9642_CCDC170	coiled-coil domain containing 170 (*CCDC170*)	M
Scaffold61	6.8E+07	0.77367	C	A	Phtr_g15498_RMND1	required for meiotic nuclear division 1 homolog (*RMND1*)	M
Scaffold61	6.8E+07	0.80087	C	T	Phtr_g6894_AKAP12	A-kinase anchoring protein 12 (*AKA12*)	M
Scaffold61	6.8E+07	0.8009	C	T	Phtr_g6894_AKAP12	A-kinase anchoring protein 12 (*AKA12*)	M
Scaffold61	6.8E+07	0.81043	G	A	Phtr_g6894_AKAP12	A-kinase anchoring protein 12 (*AKA12*)	M
Scaffold61	6.9E+07	0.7991	A	G	Phtr_g16712_LRP11	LDL receptor related protein 11 (*LRP11*)	M
Scaffold0	331489	1	G	T	Phtr_g7229_OLFML1	olfactomedin like 1 (*OLFML1*)	M
Scaffold0	335280	0.91707	A	G	Phtr_g7229_OLFML1	olfactomedin like 1 (*OLFML1*)	M
Scaffold0	428450	0.77858	C	G	Phtr_g6995_SYT9	synaptotagmin IX (*SYT9*)	M
Scaffold0	1056892	0.7305	G	T	Phtr_g20606_KIAA1549L	KIAA1549 like (*KIAA1549L*)	M
Scaffold0	1173975	1	G	T	Phtr_g17272_HIPK3	homeodomain interacting protein kinase 3 (*HIPK3*)	M
Scaffold0	1409563	0.78838	C	G	Phtr_g19449_CCDC73	coiled-coil domain containing 73 (*CCDC73*)	M
Scaffold0	1433565	0.89983	C	T	Phtr_g19449_CCDC73	coiled-coil domain containing 73 (*CCDC73*)	M
Scaffold0	3616657	0.94118	T	C	Phtr_g23581_BBOX1	gamma-butyrobetaine hydroxylase 1 (*BBOX1*)	M
Scaffold0	3942434	1	C	G	Phtr_g1219_CYB5R2	cytochrome B5 Reductase 2 (*CYB5R2*)	M
Scaffold0	3945258	0.94759	C	G	Phtr_g13521_PPFIBP2	PPFIA binding protein 2 (*PPFIBP2*)	M
Scaffold0	4213014	0.93408	G	T	Phtr_g14180_FADS1L2	fatty acid desaturase 1-like 2 (*FADS1L2*)	M
Scaffold0	4241037	0.82965	A	G	Phtr_g8765_FADS1L	fatty acid desaturase 1-like (*FADS1L*)	M
Scaffold0	4244481	0.94429	C	G	Phtr_g8765_FADS1L	fatty acid desaturase 1-like (*FADS1L*)	M
Scaffold0	4274248	1	C	T	Phtr_g25604_FADS2	fatty acid desaturase 2 (*FADS2*)	M
Scaffold0	4334858	0.88128	C	T	Phtr_g5610_OR5V1-like	Olfactory receptor 5V1-like (*OR5V1-like*)	M
Scaffold0	4352547	1	A	C	Phtr_g468_MAS1-like	MAS1 proto-oncogene, G protein-coupled receptor-like (*MAS1-like*)	M

Scaffold0	4355728	1	A	G	Phtr_g23010_MRGPRH-like	MAS-related GPR, member H-like (*MRGPRH-like*)	M
Scaffold0	4359226	1	C	A	Phtr_g16755_MRGPRH-like-2	MAS-related GPR, member H-like-2 (*MRGPRH-like-2*)	M
Scaffold0	4359229	1	T	C	Phtr_g16755_MRGPRH-like-2	MAS-related GPR, member H-like-2 (*MRGPRH-like-2*)	M
Scaffold0	4359230	1	C	A	Phtr_g16755_MRGPRH-like-2	MAS-related GPR, member H-like-2 (*MRGPRH-like-2*)	M
Scaffold0	4359232	1	G	A	Phtr_g16755_MRGPRH-like-2	MAS-related GPR, member H-like-2 (*MRGPRH-like-2*)	M
Scaffold0	4359233	1	G	C	Phtr_g16755_MRGPRH-like-2	MAS-related GPR, member H-like-2 (*MRGPRH-like-2*)	M

Supplementary Table 10. Comparison of genetic summary statistics (nucleotide diversity and Tajima's D) and demographic modelling parameter estimates when using the southern or northern assembly as reference. Genetic summary statistics are reported as means for non-overlapping 10 kb windows in each region and have been calculated for pure southern (N=10) and pure northern samples (N=7).

	**Southern assembly**	**Northern assembly**
**Summary statistics**		
**Chromosome 1**		
pi south	0.006	0.006
pi north	0.008	0.008
Tajima's D south	-0.4	-0.4
Tajima's D north	-0.9	-0.9
**Chromosome 3**		
pi south	0.006	0.006
pi north	0.006	0.006
Tajima's D south	-0.6	-0.6
Tajima's D north	-0.8	-0.7
**Chromosome 5**		
pi south	0.006	0.006
pi north	0.007	0.007
Tajima's D south	-0.3	-0.3
Tajima's D north	-0.9	-0.8

**Rest of genome**		
pi south	0.006	0.006
pi north	0.006	0.006
Tajima's D south	-1.0	-1.0
Tajima's D north	-1.0	-0.9
**Demographic modelling**		
**Chromosome 1**		
Ne_northern	1 566 413	1 565 912
Ne_southern	592 968	585 295
Ne_ancestral	273 471	269 233
split_years	1 197 770	1 183 534
**Chromosome 3**		
Ne_northern	665 708	666 873
Ne_southern	323 997	319 258
Ne_ancestral	176 903	177 353
split_years	1 302 349	1 275 961
m_e	6.53E-07	6.56E-07
**Chromosome 5**		
Ne_northern	1 519 746	1 467 626
Ne_southern	703 791	690 884
Ne_ancestral	318 141	318 360
split_years	1 218 887	1 164 125
