# Supplementary material for: Inversions maintain differences between migratory phenotypes of a songbird
Source: Nat Commun. 2023 Jan 27;14:452. doi: 10.1038/s41467-023-36167-y (PMC9883250; doi:10.1038/s41467-023-36167-y)
Supplement: Supplementary file 2 — Reporting Summary [file 41467_2023_36167_MOESM2_ESM.pdf]

nature portfolio

Corresponding author(s): Max Lundberg

Last updated by author(s): Jan 3, 2023

Reporting Summary

Nature Portfolio wishes to improve the reproducibility of the work that we publish. This form provides structure for consistency and transparency in reporting. For further information on Nature Portfolio policies, see our Editorial Policies and the Editorial Policy Checklist.

Statistics

For all statistical analyses, confirm that the following items are present in the figure legend, table legend, main text, or Methods section.

n/a	Confirmed	
☐	☒	The exact sample size (*n*) for each experimental group/condition, given as a discrete number and unit of measurement
☐	☒	A statement on whether measurements were taken from distinct samples or whether the same sample was measured repeatedly
☒	☐	The statistical test(s) used AND whether they are one- or two-sided<br>*Only common tests should be described solely by name; describe more complex techniques in the Methods section.*
☒	☐	A description of all covariates tested
☒	☐	A description of any assumptions or corrections, such as tests of normality and adjustment for multiple comparisons
☐	☒	A full description of the statistical parameters including central tendency (e.g. means) or other basic estimates (e.g. regression coefficient) AND variation (e.g. standard deviation) or associated estimates of uncertainty (e.g. confidence intervals)
☒	☐	For null hypothesis testing, the test statistic (e.g. *F*, *t*, *r*) with confidence intervals, effect sizes, degrees of freedom and *P* value noted<br>*Give P values as exact values whenever suitable.*
☒	☐	For Bayesian analysis, information on the choice of priors and Markov chain Monte Carlo settings
☒	☐	For hierarchical and complex designs, identification of the appropriate level for tests and full reporting of outcomes
☒	☐	Estimates of effect sizes (e.g. Cohen's *d*, Pearson's *r*), indicating how they were calculated

Our web collection on statistics for biologists contains articles on many of the points above.

Software and code

Policy information about availability of computer code

Data collection	No software was used in data collection
Data analysis	The following sofware were used to analyze the genomic data: HGAP4 (With Arrow polishing), Falcon unzip, hifiasm (0.15.5-r350), IrysView (2.5.1), LabelDensityCalculator v.1.3.0, Knickers v.1.5.5, bionano solve (3.2.2), Pilon (1.22), arcs (1.0.550), LINKS (1.8), GAP5 (Staden package 2.0.0.b11), minimap2 (2.13-r860, 2.22-r1101), purge haplotigs (downloaded 2019-02-15), PBJelly (PBSuite 15.8.24), kentUtils (370), assemblathon_stats.pl, busco (3.0.2), longranger (2.1.14), repeatmodeler (1.0.8), repeatmasker (4.0.7), tandem repeats finder (4.0.9), bwa (0.7.17-r1188), samtools (1.10), picardtools (2.10.3), freebayes (1.1.0), vcftools (0.1.16), bcftools (1.14), vcflib (2017-04-04), tigmint (1.1.2), SatsumaSynteny (2.0), Mummer (4.0.0rc1), cutadapt (1.823), Hisat2 (2.1.0), Stringtie (1.3.3), star (2.7.9a), gsnap (2016, trinity (2.0.2), pasa (2.0.2), stranded-coverage, augustus (3.2.3), braker (2.1.6), exonerate (2.4.0), kraken (2020-04-14), interproscan (5.30-69.0), webapollo (2.6.5), trimmomatic (0.36), blast (2.5.0+), EMBOSS Stretcher (6.6.0), bedtools (2.29.2), delly (0.9.1), graphtyper (2.7.4), plink (1.9), snpeff (5.0e), snpsift (5.0e), invclust, gIMBLE (v0.6.0), msprime (0.7), MSCM2, beagle (5.4), Sweepfinder2, selscan (1.3.0) and Popgenome (2.7.5). Custom scripts and workflows are available at https://github.com/maxlundberg/warbler_inversions.

For manuscripts utilizing custom algorithms or software that are central to the research but not yet described in published literature, software must be made available to editors and reviewers. We strongly encourage code deposition in a community repository (e.g. GitHub). See the Nature Portfolio guidelines for submitting code & software for further information.

Data

Policy information about availability of data

All manuscripts must include a data availability statement. This statement should provide the following information, where applicable:

- Accession codes, unique identifiers, or web links for publicly available datasets
- A description of any restrictions on data availability
- For clinical datasets or third party data, please ensure that the statement adheres to our policy

Raw sequence data, optical maps and de novo assemblies generated in this study are available at NCBI under bioproject PRJNA550489 (https://www.ncbi.nlm.nih.gov/bioproject/PRJNA550489). Whole-genome resequencing data used from a previous study are available in NCBI under bioproject PRJNA319295 (https://www.ncbi.nlm.nih.gov/bioproject/PRJNA319295). Source data and annotation files are available at Figshare (https://doi.org/10.6084/m9.figshare.21821328.v1).

Human research participants

Policy information about studies involving human research participants and Sex and Gender in Research.

Reporting on sex and gender	*Use the terms sex (biological attribute) and gender (shaped by social and cultural circumstances) carefully in order to avoid confusing both terms. Indicate if findings apply to only one sex or gender; describe whether sex and gender were considered in study design whether sex and/or gender was determined based on self-reporting or assigned and methods used. Provide in the source data disaggregated sex and gender data where this information has been collected, and consent has been obtained for sharing of individual-level data; provide overall numbers in this Reporting Summary. Please state if this information has not been collected. Report sex- and gender-based analyses where performed, justify reasons for lack of sex- and gender-based analysis.*
Population characteristics	*Describe the covariate-relevant population characteristics of the human research participants (e.g. age, genotypic information, past and current diagnosis and treatment categories). If you filled out the behavioural & social sciences study design questions and have nothing to add here, write "See above."*
Recruitment	*Describe how participants were recruited. Outline any potential self-selection bias or other biases that may be present and how these are likely to impact results.*
Ethics oversight	*Identify the organization(s) that approved the study protocol.*

Note that full information on the approval of the study protocol must also be provided in the manuscript.

Field-specific reporting

Please select the one below that is the best fit for your research. If you are not sure, read the appropriate sections before making your selection.

☐ Life sciences ☐ Behavioural & social sciences ☒ Ecological, evolutionary & environmental sciences

For a reference copy of the document with all sections, see nature.com/documents/nr-reporting-summary-flat.pdf

Ecological, evolutionary & environmental sciences study design

All studies must disclose on these points even when the disclosure is negative.

Study description	In this study we explore genomic differences between differentially migrating subspecies of the willow warbler Phylloscpus trochilus. We used long-read sequencing, linked-read sequencing and optical mapping to create more complete and contiguous reference genomes for each subspecies. This data allowed us to further characterize previously identifed divergent chromosome regions and detect additonal genomic differences.
Research sample	The research sample consists of genomic data from a southern (ssp. trochilus) and a northern (ssp. acredula) willow warbler. We also included genomic data from a chiffchaff (Phylloscopus collybita), to explore the evolutionary histories of the regions, and an additional willow wabler to sequence with 10x chromium to validate breakpoints. To explore highly differentiated regions we used previous whole-genome resequencing data from nine samples from each subspecies (Lundberg et al 2017. Evol. Letters.1:155-168) and also included two additional resequenced samples from each subspecies, which orginate from previous extracted samples used for SNP array genotyping (Lundberg et al 2017. Evol. Letters.1:155-168). To improve the functional annotation of differences we also included RNAseq of brain tissue from six previously collected willow warblers (Boss et al. 2016. Movement Ecology. 4, 4). Finally, we included whole-genome resequencing data from a closely related species, the dusky warbler Phylloscopus fuscatus (Bensch et al 2006. Mol Ecol. 15: 161–171).
Sampling strategy	For de novo genome assemblies of willow warblers we used one sample each for each subspecies. We chose to sequence males as it is the homogametic sex in birds and generally improve the contiguity of the assemblies (but obviously lacks the W chromosome). We further used subspecies-specific markers to verify that none of the samples represented hybrids, which would complicate the

	assembly of the divergent chromosome regions.
Data collection	Samples used for de novo genome assemblies were obtained from wild-caught birds by SB and ML
Timing and spatial scale	New samples for this study were collected during the time of autumn migration in 2017 (northern and southern willow warbler reference samples) and in autumn 2019 (chiffchaff and third willow warbler).
Data exclusions	No samples were excluded from the study
Reproducibility	Our study did not include any experiment
Randomization	Not applicable
Blinding	To the degree possible, we used the same workflow (e.g., mapping and variant calling) for different samples

Did the study involve field work? ☒ Yes ☐ No

Field work, collection and transport

Field conditions	Willow warblers and chiffchaff were caught at a stop-over-site during autumn migration. Field conditions were typical for the time of the year.
Location	Krankesjön, 15 km East of Lund, Southern Sweden.
Access & import/export	Birds were caught with permission M45-14 from Malmö/Lund Ethical Committee for Animal Research, Sweden
Disturbance	Blood was taken from the brachial vein. Following standard measurements and extraction of blood, the birds were released.

Reporting for specific materials, systems and methods

We require information from authors about some types of materials, experimental systems and methods used in many studies. Here, indicate whether each material, system or method listed is relevant to your study. If you are not sure if a list item applies to your research, read the appropriate section before selecting a response.

Materials & experimental systems

n/a	Involved in the study	
☒	☐	Antibodies
☒	☐	Eukaryotic cell lines
☒	☐	Palaeontology and archaeology
☐	☒	Animals and other organisms
☒	☐	Clinical data
☒	☐	Dual use research of concern

Methods

n/a	Involved in the study	
☒	☐	ChIP-seq
☒	☐	Flow cytometry
☒	☐	MRI-based neuroimaging

Animals and other research organisms

Policy information about studies involving animals; ARRIVE guidelines recommended for reporting animal research, and Sex and Gender in Research

Laboratory animals	No laboratory animals were used
Wild animals	New DNA samples used in this study were obtained from blood taken from males from each subspecies (ssp. trochilus and acredula). The birds were caught in mist nets and blood was extracted through puncture of the brachial vein. Following standard measurements and blood extraction, the birds were released.
Reporting on sex	We targeted male willow warblers (identified by morphology) for creating de novo assemblies. As males represent the homogametic sex in birds, they will generally generate more contiguous assemblies, particularly for the Z chromosome (but will be lacking the female-specific W chromosome). Several of the new resequencing samples and the chiffchaff sample used for de novo assembly are from females. However, in this study, where the focal regions are on autosomal chromosomes, the sex of the birds will not be important.
Field-collected samples	Blood collected from samples was stored in SET buffer or in 70 % alcohol.
Ethics oversight	Warblers were caught and blood sampled with permission M45-14 from Malmö/Lund Ethical Committee for Animal Research,

Sweden

Note that full information on the approval of the study protocol must also be provided in the manuscript.
